# Prevalence of waterpipe smoking and its associated factors among adolescents aged 12–16 years in 73 countries/territories

**DOI:** 10.3389/fpubh.2022.1052519

**Published:** 2022-11-17

**Authors:** Chuanwei Ma, Hui Yang, Min Zhao, Costan G. Magnussen, Bo Xi

**Affiliations:** ^1^Department of Epidemiology, School of Public Health, Qilu Hospital, Cheeloo College of Medicine, Shandong University, Jinan, China; ^2^Department of Nutrition and Food Hygiene, School of Public Health, Cheeloo College of Medicine, Shandong University, Jinan, China; ^3^Baker Heart and Diabetes Institute, Melbourne, VIC, Australia; ^4^Research Centre of Applied and Preventive Cardiovascular Medicine, University of Turku, Turku, Finland; ^5^Centre for Population Health Research, University of Turku and Turku University Hospital, Turku, Finland

**Keywords:** waterpipe smoking, trends, risk factors, adolescent, Global Youth Tobacco Survey (GYTS)

## Abstract

**Introduction:**

To describe the recent prevalence of, and trends in, waterpipe smoking and to examine its associated factors among adolescents aged 12–16 years in 73 countries/territories (hereafter “countries”).

**Methods:**

Data from 72 countries that had conducted a Global Youth Tobacco Survey (GYTS) in 2010–2019 and from the National Youth Tobacco Survey in the United States in 2019 were used to assess the recent prevalence of waterpipe smoking and to examine its associated factors among adolescents aged 12–16 years. Data from 38 countries that had carried out at least 2 surveys from 2000 to 2019 were used to determine trends in the prevalence of waterpipe smoking among adolescents.

**Results:**

The recent prevalence of current waterpipe smoking (on 1 day during the past 30 days) among adolescents was 6.9% (95%CI 6.4–7.5). The prevalence was highest in the European region (10.9%, 9.9–11.8) and Eastern Mediterranean region (10.7%, 9.5–11.9), but lowest in the Western Pacific region (1.9%, 1.4–2.4). The prevalence of current waterpipe smoking increased or remained unchanged in 19 (50%) of 38 countries, but decreased in the remaining 19 countries (50%). Parental smoking, closest friends' smoking, secondhand smoke exposure, tobacco advertisement exposure, not being taught the dangers of smoking, particularly cigarette smoking, were positively associated with adolescent waterpipe smoking.

**Discussion:**

Waterpipe smoking among adolescents remains a major public health issue worldwide, especially in the regions of Europe and the Eastern Mediterranean. Effective prevention and control strategies and measures are needed to curb the epidemic of adolescent waterpipe smoking.

## Introduction

Waterpipe smoking is a means of tobacco use in which the user inhales smoke from a long, soft tube that pulls smoke through the device and out of a bowl of water. Waterpipe smoking, also known as hookah, narghile, argileh, goza, chica, qalyan, shisha, and hubble-bubble (1), emerged about 500 years ago in India and Iran before spreading to other countries and is now a growing epidemic (2). There is strong evidence that waterpipe smoking is harmful to human health (3, 4). Previous studies have shown that nicotine (the major source of waterpipe addiction) (5), carbon monoxide (which can cause hypoxia and cellular respiratory disorders) (6), polycyclic hydrocarbons (well-known carcinogens or potential carcinogens) (6), and other toxicants (e.g., heavy metals, tar, and particulate matter) (7, 8) were found in mainstream waterpipe smoke. Notably, the amounts of these toxicants mentioned above may even be higher in waterpipe smoke than in cigarette smoke (9, 10). A systematic review and meta-analysis found that waterpipe smoking was associated with a heightened odds of certain types of cancer (11). Another review suggested that regular waterpipe smoking was positively associated with respiratory symptoms, lung disease, and reduced pulmonary function (12). Münzel et al. also found that waterpipe and other tobacco product (tobacco cigarettes and e-cigarettes) use might increase the burden of symptoms in patients with coronavirus disease 2019 and cause more serious health consequences (13).

The harmful effects of waterpipe smoking are not restricted to adults, with adverse health effects such as acute lung infection and injury, carbon monoxide poisoning, and subclinical consequences (e.g., lung function decline, oral and systemic genotoxicity, and the alteration of vascular and hemodynamic functions) found among adolescents (14). Abbadi et al. found that waterpipe nicotine dependence was associated with depressive symptoms among adolescents (15). Alomari et al. reported that waterpipe smoking could inhibit the brain-derived neutrophic factor (which is the pivotal for neuronal survival, migration, dendritic arborization, synaptogenesis and differentiation) among adolescents (16). In addition, Ramôa et al. called on health care and dental care professionals to focus on the increasing popularity of waterpipe smoking among adolescents due to the potential oral hazards (17).

Historically, waterpipe smoking predominated in older men in certain countries. However, waterpipe smoking has become increasingly popular among young adults and has spread to more countries. Salloum et al. found that the prevalence of waterpipe smoking (defined as ≥1 day during the past 30 days) among young adults aged 18–29 years was 60.7% in Egypt, 67.7% in Jordan, and 63.1% in Palestine in 2016 (18). A recent systematic review found that waterpipe smoking was prevalent among university students in some Arab countries, especially in the Kingdom of Saudi Arabia (~36.4%) (19). In addition, waterpipe smoking is widespread among young adults in South Africa (20), Germany (21), and Syria (22). However, to our knowledge, no previous study has assessed the global prevalence of, and trends in, waterpipe smoking among adolescents aged <18 years, particularly in low- and middle-income countries.

In this study, using the most recent data from the Global Youth Tobacco Surveys (GYTS) collected from 2010 to 2019 and a similar survey conducted in the U. in 2019, we aimed to assess the prevalence of, and trends in, waterpipe smoking among adolescents aged 12–16 years in 73 countries/territories (hereafter “countries”). We also examined the potential factors associated with adolescent waterpipe smoking.

## Methods

### Study participants

The most recent data (collected from 2010 to 2019) on waterpipe smoking among adolescents aged 12–16 years were extracted from the self-administered, nationally representative, school-based, and cross-sectional GYTS conducted in 72 countries, which has been described in previous published studies based on the GYTS (23, 24). The protocol of GYTS was developed by the WHO and the US Centers for Disease Control and Prevention (CDC). All countries that conducted the GYTS followed a two-stage sampling strategy. The first stage involved randomly selecting schools in each country, and the second stage involved randomly selecting classes from all selected schools. All students in the selected classes were eligible to complete a standardized, anonymous questionnaire voluntarily. All GYTS were approved by each the respective ethical boards in each country. More details of the GYTS are available from the US CDC website (https://nccd.cdc.gov/GTSSDataSurveyResources/Ancillary/Documentation.aspx?SUID=1&DOCT=1).

For the US, we used data from the National Youth Tobacco Survey (NYTS) performed in 2019, which is similar with GYTS. The NYTS is performed among a representative sample that surveys tobacco use and related factors among U.S. adolescents. The questionnaire items and responses on waterpipe smoking and other related factors are identical between the NYTS and the GYTS. Further details of the NYTS are available from the U.S. CDC website (https://www.cdc.gov/tobacco/data_statistics/index.htm). Both the GYTS and the NYTS are ongoing surveys, with data collection repeated at regular intervals. Verbal or written consent was obtained from both the adolescent participants and their parents/guardians in both surveys.

To assess the recent prevalence of adolescent waterpipe smoking and its associated factors for this study, we used the most recent data that was available from the GYTS conducted in 2010–2019 in 72 countries, and the NYTS that was conducted in 2019 in the US. We also assessed the trends in the prevalence of adolescent waterpipe smoking from 2000 to 2019 based on data from 38 countries that had completed at least two GYTS surveys during this time period. The flowchart of exclusion and inclusion of participating countries is shown in Supplementary Figure S1.

### Definition of waterpipe smoking

Current waterpipe smoking was defined as using waterpipe on ≥1 day during the past 30 days. Adolescents were asked to respond to the survey question “During the past 30 days, on how many days did you smoke waterpipe?,” with the following frequency options for response: “0 days,” “1 to 2 days,” “3 to 5 days,” “6 to 9 days,” “10 to 19 days,” “20 to 29 days,” and “All 30 days.” Ever tried or experimented with waterpipe smoking was assessed by the survey question “Have you ever tried or experimented with waterpipe smoking, even one or two puffs?,” with options for response of “Yes” and “No.” The location of last place of waterpipe smoking during the past 30 days was assessed by the survey question “The last time you smoked waterpipe during the past 30 days, where did you smoke it?,” with the response options of “At home,” “At a coffee shop,” “At a restaurant,” “At a bar or club,” and “Other,” The age that waterpipe smoking was initiated was assessed by the survey question “How old were you when you first tried smoking waterpipe?,” with the response options of “7 years old or younger,” “8 or 9 years old,” “12 or 13 years old,” “14 or 15 years old,” and “16 years old.” It should be noted that different countries have different waterpipe names (e.g., European countries: shisha; Arab countries: hookah and nargila (nargulia/nargile/nargileh), narguileh; other countries: hubble-bubble, gudugudaa, chicha), with these terms used in place of “waterpipe,” as appropriate.

### Potential associated factors

Current cigarette smoking was assessed using the survey question: “During the past 30 days, on how many days did you smoke cigarettes?” and defined as a response of smoking a cigarette on ≥1 day during the past 30 days. Secondhand smoke exposure was assessed with the following three questions: “During the past 7 days, on how many days has anyone smoked inside your home, in your presence?”; “During the past 7 days, on how many days has anyone smoked in your presence, inside any enclosed public place, other than in your home?”; and “During the past 7 days, on how many days has anyone smoked in your presence, at any outdoor public place?” with the responses to these questions combined to define exposure to secondhand smoke at home or in enclosed or outdoor places on at least 1 day during the past 7 days. Parental smoking status was assessed using the survey question “Do your parents smoke tobacco?,” with the answers of “None,” “Father only,” “Mother only,” and “Both.” Closest friends' smoking status was assessed using the survey question “Do any of your closest friends smoke tobacco?,” with response options of “None of them,” “Some of them,” “Most of them,” and “All of them.” Whether or not being taught dangers of smoking status was assessed by the survey question “During the past 12 months, were you taught in any of your classes about the dangers of tobacco use?,” with response options of “Yes” and “No.” Exposure to tobacco advertisements was assessed from three questions: “During the past 30 days, did you see any people using tobacco on TV, in videos, or movies?,” “During the past 30 days, did you see any advertisements or promotions for tobacco products at points of sale?,” and “Do you have something (for example, t-shirt, pen, backpack) with a tobacco product brand logo on it?” with exposure defined as a response of exposure to tobacco advertisements *via* at least one of the above-mentioned avenues. Each country's income level was defined based on the World Bank classification according to the survey year of the GYTS.

### Statistical analysis

Prevalence estimates and 95% confidence intervals (CI) of current and ever tried or experimented with waterpipe smoking were calculated using the sampling weights, strata, primary sampling units provided in the GYTS dataset in each country. The weighted prevalence at national level was calculated based on the original sampling weights, and the overall and subgroup (sex, age group, regional, and other) estimates were calculated based on the rescaled weights, with consideration of the sample size of each country. Chi-square test was used to test the differences in the prevalence estimates between groups (sex, age group, WHO region, World Bank income category, cigarette smoking status, secondhand smoke exposure status, and parental smoking status). Poisson regression analyses were used to examine the trends in the prevalence of waterpipe smoking, with consideration of all available GYTS data in all survey years for each country. Multivariable logistic regression analyses were used to examine the association between waterpipe smoking and potential influencing factors (sex, age group, parental smoking status, smoking status of closest friends, cigarette smoking status, secondhand smoke exposure status, tobacco advertisement exposure status, being taught dangers of smoking status, World Bank income category, and survey year). All analyses were performed using SAS 9.4 (SAS Institute, Cary, NC, US). A two-sided *P* < 0.05 was considered statistically significant.

## Results

### Participant characteristics

In this study, 335,062 adolescents aged 12–16 years (boys: 51.1%) from the 73 included countries had data on waterpipe smoking in 2010–2019. Of these 73 countries, 11 (15.1%) were located in the African region, 17 (23.3%) in the American region, 22 (30.1%) in the Eastern Mediterranean region, 18 (24.7%) in the European region, 2 (2.7%) in the South-East Asian region, and 4 (5.5%) in the Western Pacific region (Supplementary Table S1).

### Prevalence of and trends in waterpipe smoking

**Figure 1:**
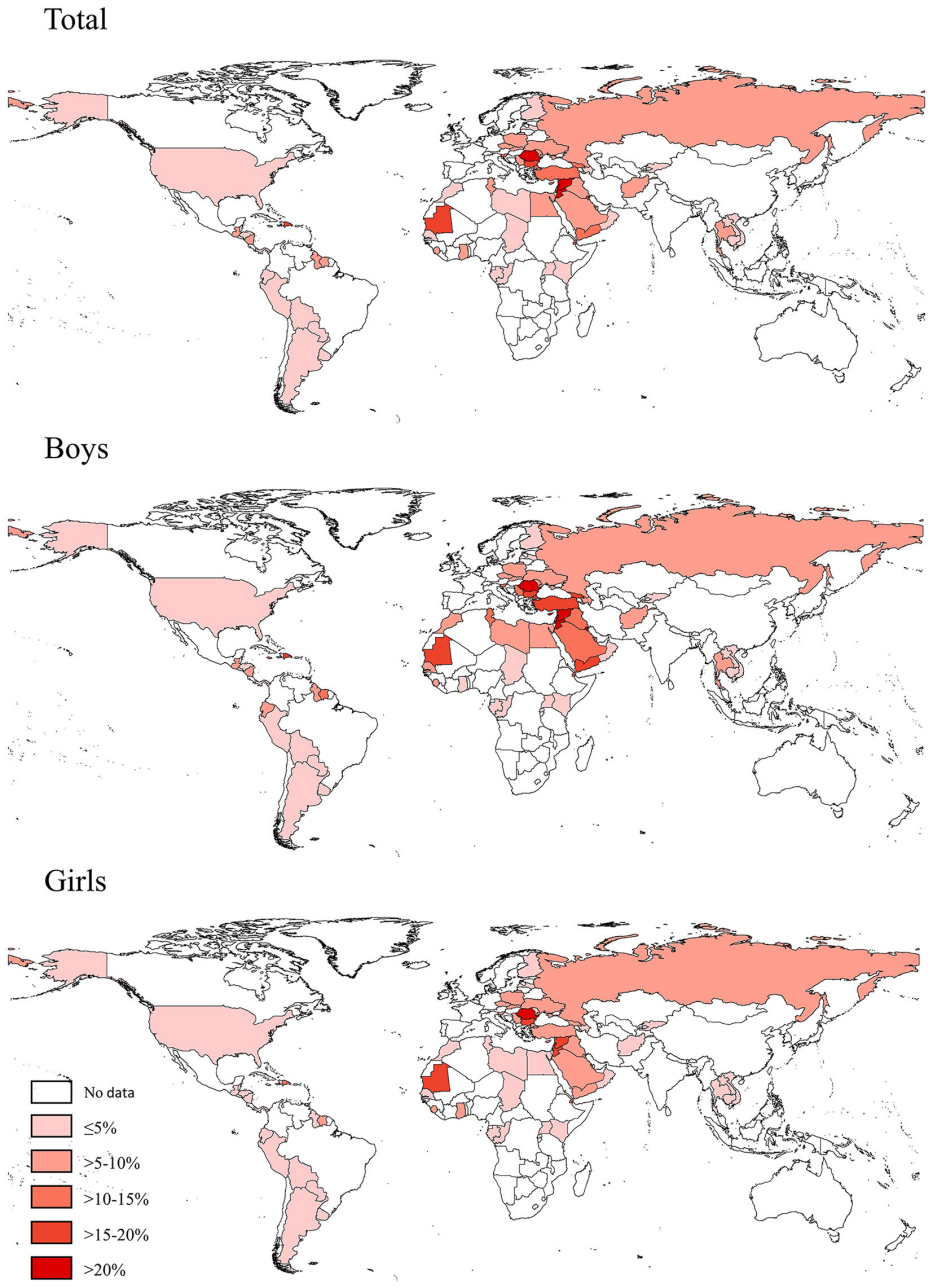
Prevalence of waterpipe smoking (on ≥1 day during the past 30 days) among adolescents aged 12–16 years in 73 countries/territories in 2010–2019.

Based on the most recent data conducted in 73 countries in 2010–2019, 6.9% (95% CI 6.4–7.5) of adolescents aged 12–16 years reported to have used a waterpipe on ≥1 day during the past 30 days, 3.0% (2.7–3.2) on ≥3 days, and 1.6% (1.4–1.8) on ≥6 days. The prevalence of current waterpipe smoking (on ≥1 day during the past 30 days) was higher among boys (8.5%, 7.7–9.3) and older adolescents aged 15–16 years (8.7%, 7.8–9.7) than among girls (5.3%, 4.7–5.8) and those aged 12–14 years (5.8%, 5.3–6.3). Sex and age differences remained when waterpipe smoking was ≥3 or ≥6 days during the past 30 days (Table 1). The prevalence of current waterpipe smoking varied significantly across countries even within the same WHO region (Figure 1 and Supplementary Table S2). The prevalence was highest in the European and Eastern Mediterranean regions and lowest in the Western Pacific region. The prevalence was higher in lower-middle income and upper-middle income countries than in low-income and high-income countries. The prevalence was approximately seven times higher among current cigarette users compared with those who did not currently smoke cigarettes, and was three times higher among adolescents who indicated they were exposed to secondhand smoke compared with those not exposed. The prevalence among adolescents whose fathers only, mothers only, and both smoked tobacco was higher than those whose parents did not smoke. Similar patterns were observed in other frequency categories of waterpipe smoking (≥3 or ≥6 days during the past 30 days) (Table 1). We also assessed the prevalence of ever tried or experimented with waterpipe using the most recent data (2010–2019) in 70 countries (Supplementary Tables S3, S4) and the prevalence was much higher (>10%) in most countries (49/70).

**Table 1:** Prevalence of waterpipe smoking among adolescents aged 12–16 years by use frequency, sex, age group, WHO region, World Bank income category, cigarette use, secondhand smoke exposure, and parental smoking, 2010–2019.

**Group**	**No. of countries**	≥**1 day**			≥**3 days**			≥**6 days**		
		**Total**	**Boys**	**Girls**	**Total**	**Boys**	**Girls**	**Total**	**Boys**	**Girls**
**Total**	73	6.9 (6.4–7.5)	8.5 (7.7–9.3)	5.3 (4.7–5.8)*	3.0 (2.7–3.2)	3.9 (3.5–4.3)	1.9 (1.7–2.2)*	1.6 (1.4–1.8)	2.2 (1.9–2.5)	0.9 (0.8–1.1)*
**Age group**										
12–14 years	73	5.8 (5.3–6.3)	6.9 (6.3–7.6)	4.8 (4.2–5.4)*	2.4 (2.1–2.7)	3.1 (2.7–3.5)	1.7 (1.4–1.9)*	1.2 (1.0–1.3)	1.6 (1.4–1.9)	0.8 (0.6–0.9)*
15–16 years	73	8.7 (7.8–9.7)	11.1 (9.8–12.3)	6.1 (5.2–7.0)*	3.9 (3.4–4.4)	5.2 (4.5–5.9)	2.4 (2.0–2.9)*	2.2 (1.8–2.6)	3.1 (2.5–3.6)	1.2 (0.9–1.5)*
*P*-value		<0.0001	<0.0001	0.0041	<0.0001	<0.0001	0.0007	<0.0001	<0.0001	<0.0001
**WHO region**										
Africa	11	4.2 (3.3–5.0)	4.5 (3.2–5.7)	3.9 (3.0–4.8)	1.8 (1.3–2.4)	2.0 (1.2–2.9)	1.6 (1.1–2.2)	1.0 (0.7–1.3)	1.0 (0.6–1.4)	1.0 (0.6–1.4)
Americas	17	4.2 (3.2–5.2)	4.4 (3.3–5.5)	4.0 (2.9–5.1)	1.6 (1.1–2.2)	1.8 (1.2–2.5)	1.5 (0.9–2.0)	0.9 (0.5–1.3)	1.0 (0.5–1.5)	0.8 (0.4–1.2)
Eastern Mediterranean	22	10.7 (9.5–11.9)	13.9 (12.3–15.6)	7.3 (6.1–8.6)*	4.4 (3.8–5.0)	6.5 (5.7–7.3)	2.2 (1.8–2.7)*	2.4 (2.1–2.8)	3.7 (3.1–4.3)	1.1 (0.8–1.4)*
Europe	18	10.9 (9.9–11.8)	12.9 (11.7–14.0)	8.8 (7.9–9.8)*	6.2 (5.5–6.9)	7.2 (6.3–8.0)	5.2 (4.4–6.0)*	2.4 (2.1–2.7)	3.2 (2.8–3.6)	1.5 (1.3–1.8)*
South-East Asia	2	5.4 (3.7–7.2)	7.9 (5.5–10.3)	2.9 (1.3–4.5)*	2.1 (1.3–3.0)	3.3 (1.7–4.9)	1.0 (0.4–1.6)*	1.4 (0.8–1.9)	2.5 (1.4–3.6)	0.2 (0.0–0.5)*
Western Pacific	3	1.9 (1.4–2.4)	2.5 (1.7–3.2)	1.3 (0.8–1.9)*	0.5 (0.3–0.7)	0.7 (0.4–1.0)	0.4 (0.2–0.6)	0.3 (0.1–0.4)	0.2 (0.1–0.4)	0.3 (0.1–0.5)
*P*-value		<0.0001	<0.0001	<0.0001	<0.0001	<0.0001	<0.0001	<0.0001	<0.0001	0.0049
**World Bank income**										
Low income	10	4.6 (3.8–5.4)	5.2 (4.0–6.4)	4.0 (3.1–4.9)	1.9 (1.5–2.3)	2.3 (1.5–3.0)	1.5 (1.0–2.1)	1.1 (0.7–1.5)	1.3 (0.8–1.8)	0.9 (0.4–1.4)
Lower-Middle income	23	8.1 (7.1–9.1)	10.5 (9.1–11.9)	5.6 (4.7–6.6)*	3.1 (2.7–3.6)	4.5 (3.8–5.2)	1.7 (1.3–2.0)*	1.6 (1.3–1.8)	2.3 (1.8–2.8)	0.8 (0.6–1.0)*
Upper-Middle income	23	7.3 (6.3–8.4)	8.7 (7.5–10.0)	5.9 (4.9–7.0)*	3.5 (2.9–4.1)	4.4 (3.5–5.2)	2.6 (2.0–3.1)*	1.8 (1.4–2.2)	2.5 (1.9–3.1)	1.1 (0.8–1.5)*
High income	17	4.7 (4.1–5.2)	5.6 (4.7–6.4)	3.8 (3.2–4.4)*	2.0 (1.7–2.3)	2.5 (2.0–3.0)	1.5 (1.1–1.8)*	1.4 (1.1–1.5)	1.9 (1.4–2.3)	0.9 (0.6–1.1)*
*P*-value		<0.0001	<0.0001	0.0018	<0.0001	<0.0001	0.0002	0.0065	0.038	0.34
**Cigarette smoking**										
Yes	73	30.1 (25.9–34.4)	32.8 (28.7–36.8)	25.3 (20.1–30.5)*	15.8 (13.3–18.4)	17.7 (15.1–20.4)	12.3 (9.1–15.4)*	9.2 (7.6–10.9)	10.8 (8.9–12.6)	6.4 (4.5–8.2)*
No	73	4.2 (3.8–4.7)	4.9 (4.3–5.5)	3.6 (3.2–4.1)*	1.5 (1.3–1.7)	1.9 (1.6–2.2)	1.1 (0.9–1.3)*	0.7 (0.6–0.9)	1.0 (0.8–1.2)	0.5 (0.4–0.6)*
*P*-value		<0.0001	<0.0001	<0.0001	<0.0001	<0.0001	<0.0001	<0.0001	<0.0001	<0.0001
**Secondhand smoke exposure**										
Yes	73	9.8 (9.0–10.7)	12.3 (11.2–13.5)	7.3 (6.4–8.1)*	4.3 (3.8–4.7)	5.8 (5.1–6.5)	2.7 (2.3–3.1)*	2.3 (2.0–2.6)	3.3 (2.8–3.8)	1.3 (1.1–1.5)*
No	73	3.0 (2.6–3.4)	3.5 (3.0–3.9)	2.6 (2.0–3.1)*	1.2 (1.0–1.4)	1.5 (1.2–1.7)	0.9 (0.7–1.2)*	0.6 (0.5–0.7)	0.7 (0.6–0.9)	0.4 (0.3–0.6)*
*P*-value		<0.0001	<0.0001	<0.0001	<0.0001	<0.0001	<0.0001	<0.0001	<0.0001	<0.0001
**Parental smoking**										
Both	46	23.1 (20.6–25.6)	26.3 (22.7–30.0)	19.6 (17.0–22.2)*	12.3 (10.4–14.2)	14.9 (11.8–17.9)	9.5 (7.7–11.2)*	7.0 (5.4–8.6)	9.7 (6.9–12.4)	4.1 (2.8–5.4)*
Father only	46	11.3 (10.0–12.6)	14.9 (12.6–17.2)	8.0 (6.8–9.1)*	4.4 (3.7–5.0)	6.4 (5.2–7.6)	2.5 (2.0–3.0)*	2.2 (1.8–2.6)	3.3 (2.6–4.0)	1.1 (0.8–1.5)*
Mother only	46	23.9 (19.6–28.2)	30.7 (23.3–38.1)	17.4 (13.4–21.5)*	9.4 (7.5–11.3)	11.2 (8.3–14.1)	7.7 (5.3–10.1)*	4.8 (3.5–6.0)	6.5 (4.5–8.5)	3.1 (1.7–4.6)*
Neither	46	6.1 (5.5–6.6)	7.7 (6.8–8.6)	4.4 (3.9–4.9)*	2.6 (2.2–2.9)	3.5 (3.0–4.1)	1.6 (1.3–1.9)*	1.3 (1.1–1.5)	1.9 (1.6–2.3)	0.6 (0.5–0.8)*
*P*-value		<0.0001	<0.0001	<0.0001	<0.0001	<0.0001	<0.0001	<0.0001	<0.0001	<0.0001

Data are presented as % (95% CI).

WHO, World Health Organization.

* There was a statistically significant difference between sexes.

Based on the most recent data conducted in 52 countries in 2010–2019, 26.9% (23.4–30.4) of adolescents reported their initially use of waterpipe occurred before age 9 years, 39.5% (36.4–42.6) from age 10–13 years, and 34.2% (30.8–37.5) from age 14–16 years. More details of the distributions of age of initial waterpipe smoking by country, sex, WHO region, World Bank income category, current cigarette use status, secondhand smoke exposure status, and parental smoking status are shown in Supplementary Tables S5, S6.

Based on the most recent data from 48 countries in 2010–2019, 37.9% (35.4–40.3) of adolescents reported the last place that they used a waterpipe during the past 30 days was at home, 17.7% (16.0–19.3) at coffee shops, 9.9% (8.1–11.7) at a restaurant, 7.0% (5.8–8.1) at a bar or club, and 27.6% (25.3–30.0) at other places (Table 2). Boys tended to last use a waterpipe at coffee shops compared with girls, while for girls their last use of waterpipe more often occurred at home. The proportions of last waterpipe smoking at a restaurant, bar or club, and other places did not differ significantly between boys and girls in most countries. Young adolescents aged 12–14 years tended to last use waterpipe at home compared with older adolescents aged 15–16 years, while older adolescents aged 15–16 years last use of a waterpipe more often occurred at bars or clubs. The proportion of the last places to use a waterpipe during the past 30 days varied significantly by country, WHO region, and World Bank income category (Table 2 and Supplementary Tables S7, S8).

**Table 2:** Proportions of the last place of waterpipe smoking during the past 30 days among adolescent waterpipe users by sex, age group, WHO region, World Bank income category, cigarette use, secondhand smoke exposure, and parental smoking, 2010–2019.

**Group**	**No. of countries**	**Home**	**Coffee shop**	**Restaurant**	**Bar or club**	**Other places**
**Total**	48	37.9 (35.4–40.3)	17.7 (16.0–19.3)	9.9 (8.1–11.7)	7.0 (5.8–8.1)	27.6 (25.3–30.0)
**Sex**						
Boys	48	33.9 (31.0–36.8)	21.2 (18.9–23.5)	9.0 (7.1–10.9)	6.8 (5.2–8.5)	29.1 (25.9–31.2)
Girls	48	43.9 (40.0–47.9)	12.2 (10.2–14.2)	11.3 (7.5–15.0)	7.2 (5.2–9.2)	25.4 (21.7–29.1)
*P*-value		<0.0001	<0.0001	0.27	0.79	0.14
**Age group**						
12–14 years	48	41.0 (37.5–44.5)	17.0 (14.4–19.5)	10.2 (7.8–12.5)	4.9 (3.9–5.9)	27.0 (23.9–30.1)
15–16 years	48	34.3 (30.9–37.6)	18.5 (15.6–21.3)	9.6 (7.1–12.0)	9.4 (7.4–11.4)	28.3 (24.8–31.7)
*P*-value		0.0053	0.48	0.71	<0.0001	0.57
**WHO region**						
Africa	7	35.4 (27.4–43.4)	24.1 (17.5–30.6)	13.1 (8.2–18.0)	10.0 (7.2–12.7)	17.4 (8.5–26.3)
Americas	9	27.8 (22.9–32.6)	10.1 (6.9–13.4)	4.2 (2.3–6.1)	14.9 (10.8–19.0)	42.9 (37.7–48.2)
Eastern Mediterranean	21	41.7 (38.4–44.9)	19.9 (17.6–22.1)	11.3 (8.7–14.0)	2.9 (2.2–3.6)	24.2 (21.4–27.0)
Europe	7	28.5 (24.5–32.6)	24.2 (19.8–28.6)	10.0 (7.8–12.1)	16.4 (13.9–18.8)	21.0 (17.3–24.6)
South-East Asia	1	40.9 (30.5–51.2)	3.8 (0.4–7.2)	4.9 (1.3–8.5)	14.3 (7.4–21.2)	36.1 (27.4–44.9)
Western Pacific	3	20.9 (13.9–27.8)	22.5 (13.8–31.2)	18.7 (14.3–23.2)	12.2 (5.5–18.9)	25.7 (16.8–34.4)
*P*-value		<0.0001	<0.0001	<0.0001	<0.0001	<0.0001
**World Bank income**						
Low income	7	46.9 (41.4–52.3)	23.8 (19.0–28.6)	9.7 (6.8–12.7)	3.3 (2.5–4.1)	16.3 (13.8–18.8)
Lower-Middle income	18	37.1 (33.4–40.7)	21.2 (18.7–23.6)	14.5 (11.3–17.8)	4.5 (3.5–5.5)	22.7 (19.4–26.1)
Upper-Middle income	15	38.8 (34.7–42.8)	12.8 (10.2–15.4)	4.8 (3.5–6.2)	10.4 (8.1–12.8)	33.2 (29.6–36.8)
High income	8	34.6 (30.1–39.1)	17.0 (12.2–21.7)	4.5 (3.2–5.8)	7.1 (4.9–9.2)	36.8 (29.0–44.7)
*P*-value		0.19	<0.0001	<0.0001	<0.0001	<0.0001
**Cigarette smoking**						
Yes	48	29.8 (25.5–34.0)	23.6 (19.5–27.7)	8.3 (4.7–11.8)	8.4 (6.0–10.8)	30.0 (26.3–33.7)
No	48	40.7 (38.0–43.5)	15.4 (13.3–17.5)	9.9 (7.9–11.8)	6.4 (5.0–7.8)	27.6 (24.2–31.1)
*P-*value		<0.0001	0.0002	0.45	0.12	0.41
**Secondhand smoke exposure**						
Yes	48	38.0 (35.4–40.6)	18.3 (16.3–20.2)	9.8 (7.8–11.8)	6.7 (5.4–7.9)	27.2 (24.9–29.5)
No	48	37.8 (33.5–42.1)	15.2 (12.0–18.4)	10.2 (7.7–12.6)	8.1 (5.8–10.5)	28.7 (24.1–33.3)
*P*-value		0.94	0.11	0.82	0.25	0.51
**Parental smoking**						
Both	28	47.9 (41.9–54.0)	16.1 (12.7–19.6)	8.4 (4.8–12.1)	4.2 (2.6–5.7)	23.4 (18.6–28.1)
Father only	28	42.4 (38.0–46.8)	12.2 (9.9–14.5)	9.1 (5.9–12.4)	6.3 (4.3–8.3)	29.9 (25.9–34.0)
Mother only	28	33.6 (23.6–43.5)	11.8 (6.1–17.4)	12.0 (4.9–19.0)	4.9 (2.0–7.9)	37.7 (23.6–51.9)
Neither	28	38.3 (34.9–41.7)	16.0 (13.8–18.2)	9.9 (7.8–11.9)	4.9 (3.6–6.2)	30.9 (27.2–34.7)
*P*-value		0.029	0.039	0.82	0.28	0.099

Data are presented as % (95% CI).

WHO, World Health Organization.

In this study, 38 countries had conducted ≥2 surveys from 2000 to 2019. 504,686 adolescents aged 12–16 years were included to assess trends in the prevalence of current waterpipe smoking from 2000 to 2019. The trends in the prevalence of waterpipe smoking varied across countries (Supplementary Table S9). From 2000 to 2019, the prevalence of waterpipe smoking decreased in 19 (50.0%) countries, increased in 18 (47.4%) countries, and remained unchanged in 1 (2.6%) country (Table 3). In addition, the overall and subgroup (e.g., sex, WHO region, World Bank income category, cigarette smoking status, secondhand smoke exposure status, and parental smoking status) prevalence of waterpipe smoking (calculated as per 5 calendar-years) remained unchanged from 2000 to 2019 (Supplementary Table S10).

**Table 3:** Proportions of countries with upward, downward, and unchanged trends in waterpipe smoking (on ≥ 1 day during the past 30 days) among adolescents aged 12–16 years from 2000 to 2019.

**Group**	**No. of countries**	**Total**			**Boys**			**Girls**		
		Downward**	Upward**	Unchanged*	Downward**	Upward**	Unchanged*	Downward**	Upward**	Unchanged*
**Total**	38	50.0	47.4	2.6	52.6	42.1	5.3	47.4	47.4	5.3
**WHO region**										
Americas	6	67.0	33.0	0.0	66.7	16.7	16.7	66.7	33.3	0.0
Eastern Mediterranean	20	45.0	50.0	5.0	50.0	45.0	5.0	45.0	45.0	10.0
Europe	11	54.6	45.5	0.0	54.6	45.5	0.0	45.5	54.6	0.0
South-East Asia	1	0.0	100.0	0.0	0.0	100.0	0.0	0.0	100.0	0.0
**World Bank income**										
Low income	4	50.0	50.0	0.0	50.0	50.0	0.0	50.0	25.0	25.0
Lower-Middle income	14	57.1	42.9	0.0	57.1	42.9	0.0	57.1	42.9	0.0
Upper-Middle income	9	44.4	44.4	11.1	55.6	44.4	0.0	33.3	55.6	11.1
High income	11	45.5	54.5	0.0	45.5	36.4	18.2	45.5	54.6	0.0

Data are presented as %.

WHO, World Health Organization.

Poisson regression analysis was used to examine the trend in prevalence across all survey years (detailed results are listed in Supplementary Table S9, **P for trend < 0.05; *P for trend >0.05).

### Association between waterpipe smoking and potential factors

As shown in Table 4, boys (vs. girls, OR = 1.31, 95% CI = 1.16–1.48), older adolescents aged 15–16 years (vs. younger adolescents aged 12–14 years, OR = 1.22, 95% CI = 1.07–1.38), parental smoking (father alone: OR = 1.51, 95% CI = 1.33–1.71; mother alone: OR = 2.76, 95% CI = 2.09–3.64; both: OR = 2.26, 95% CI = 1.91–2.66), closest friends' smoking (some: OR = 2.10, 95% CI = 1.84–2.40; most: OR = 2.74, 95% CI = 2.34–3.20; all: OR = 4.12, 95% CI = 3.16–5.37), current cigarette smoking (vs. not, OR = 4.76, 95% CI = 4.10–5.53), secondhand smoke exposure (vs. not, OR = 1.58, 95% CI = 1.39–1.80), tobacco advertisement exposure (vs. not, OR = 1.19, 95% CI = 1.07–1.33), and not being taught dangers of smoking (vs. being taught, OR = 1.23, 95% CI = 1.10–1.38) were associated with higher odds of adolescents waterpipe smoking. Subgroup analyses by sex, national income level and current cigarette smoking status showed similar results (Supplementary Tables S11–S13).

**Table 4:** Factors associated with waterpipe smoking (on ≥1 day during the past 30 days) among adolescents aged 12–16 years in 73 countries/territories, 2010–2019.

**Variable**	**Prevalence (%)**	**β**	**OR (95% CI)**
**Sex**			
Girls	5.3		1.00
Boys	8.5	0.273	1.31 (1.16–1.48)
**Age group**			
12–14 years	5.8		1.00
15–16 years	8.7	0.197	1.22 (1.07–1.38)
**Parental smoking status**			
Neither	6.1		1.00
Father only	11.3	0.411	1.51 (1.33–1.71)
Mother only	23.9	1.014	2.76 (2.09–3.64)
Both	23.1	0.814	2.26 (1.91–2.66)
**Smoking status of closest friends**			
None	4.2		1.00
Some	12.1	0.741	2.10 (1.84–2.40)
Most	21.0	1.006	2.74 (2.34–3.20)
All	36.9	1.416	4.12 (3.16–5.37)
**Cigarette smoking**			
No	4.2		1.00
Yes	30.1	1.561	4.76 (4.10–5.53)
**Secondhand smoke exposure**			
No	3.0		1.00
Yes	9.8	0.459	1.58 (1.39–1.80)
**Tobacco advertisements exposure**			
No	4.4		1.00
Yes	7.7	0.174	1.19 (1.07–1.33)
**Being taught about dangers of smoking**			
Yes	6.5		1.00
No	7.9	0.210	1.23 (1.10–1.38)
**World Bank income**			
Low income	4.6		1.00
Lower-Middle income	8.1	0.565	1.76 (1.39–2.22)
Upper-Middle income	7.3	1.716	5.56 (4.25–7.28)
High income	4.7	0.164	1.18 (0.90–1.54)
**Survey year**			
2016–2019	5.6		1.00
2010–2015	8.4	0.437	1.55 (1.32–1.81)

All variables listed in the table were introduced into logistic regression models.

OR, odds ratio; CI, confidence interval.

## Discussion

Based on the most recent GYTS data (2010–2019) from 73 countries, 6.9% (6.4–7.5) of adolescents aged 12–16 years reported waterpipe smoking on ≥1 day during the past 30 days. The prevalence was highest in the European and Eastern Mediterranean regions. The most common places of last use of waterpipe were home (37.9%) and coffee shop (17.7%). In addition, the prevalence of current waterpipe smoking increased or remained unchanged in 19 (50%) of 38 countries. We found that the prevalence was higher in boys (vs. girls) and older adolescents (vs. younger ones). Parental smoking, closest friends' smoking, current cigarette smoking, secondhand smoke exposure, and not being taught the dangers of smoking were positively associated with waterpipe smoking among adolescents.

In this study, the overall prevalence of current waterpipe smoking was highest in Romania (36.9%) and lowest in Chad (0.7%) and Peru (0.7%), with more than 30% (22/73) of included countries having a prevalence of >10%. Previous studies in some specific countries also showed large differences in the prevalence of current waterpipe smoking, e.g., 46.1% in 2019 among Iraq male adolescents aged 15–18 years (25), 59.1% in 2018 among Jordan adolescents (mean age 14.6 years) (26), and 2.5% in 2017 among US middle and high school students aged 10–17 years (27). Since the age distributions of these previous studies were different from our study, direct comparison is not suitable. Nevertheless, these findings suggest that the prevalence of waterpipe smoking is already high among adolescents in many countries, underlying the need for more effort to prevent and control waterpipe smoking among adolescents.

To our knowledge, this is the first study to assess the prevalence of waterpipe smoking according to different use frequencies. We found that the prevalence was much lower when based on ≥3 days (3.0%) and ≥6 days (1.6%) vs. ≥1 day (6.9%) during the past 30 days, which was consistent with the prevalence of cigarette smoking based on different frequencies of use (28). This suggests that most waterpipe users (on ≥1 day) in adolescents are experimental. However, it is suggested that more than 10% of irregular smokers would become regular smokers after 1 year of follow-up (29). We also found that most adolescents first tried smoking a waterpipe before 13 years old. Given the addictive nature of waterpipe smoking, health education programs and regulatory frameworks to prevent waterpipe smoking among adolescents at an early age should be given priority.

We found that the prevalence of waterpipe smoking was highest in the Eastern Mediterranean and European regions. A systematic review that included 129 studies from 68 countries also reported that the prevalence of waterpipe smoking among adults was highest in the Eastern Mediterranean region, and the prevalence was also higher among youth in Eastern Mediterranean and European regions (although the prevalence estimates were mainly based on data collected before 2010) (30). Salloum et al. also reported that the prevalence of waterpipe smoking among young adults (18–29 years) in three Eastern Mediterranean countries was high (Egypt: 60.7%; Jordan: 67.7%; Palestine: 63.1%) (18). The main reason might be that waterpipe smoking is popular at social gatherings and has become ingrained in culture in these countries and regions (31). These findings suggest the need for more effective strategies and measures aimed at waterpipe smoking in Eastern Mediterranean and European countries.

Our study showed that the most common place for last waterpipe smoking was at home (37.9%), especially for girls and younger adolescents (aged 12–14 years), followed by coffee shops (17.7%). One study reported that the proportion of waterpipe smoking in coffee shops among young adults (18–29 years) was highest in three Eastern Mediterranean countries (Egypt: 74.0%, Palestine: 44.8%, Jordan: 43.0%) (18). Another study reported that home, friends' houses, coffee shops, and hookah bars were the most popular places for US young adult users aged 18–24 years (32). Widespread waterpipe cafe culture in some countries coupled with peer pressure might cause more adolescents to smoke waterpipe at coffee shops. Our data might be useful to inform policymakers of where targeted prevention might be directed to prevent adolescents from initiating or regularly smoking of a waterpipe.

Worryingly, the prevalence of current waterpipe smoking increased or remained unchanged in 50% (19/38) of countries. Similar unchanged trends were found in German adolescents aged 11–17 years from 2014 to 2017 (21) and Great Britain adolescents aged 11–18 years from 2013 to 2016 (33). Although a previous study showed a downward trend of waterpipe smoking among US adolescents aged 10–17 years from 2011 to 2017 (27), we observed a slight upward trend in waterpipe smoking among US adolescents from 2011 to 2019. The prevalence of waterpipe smoking did not decrease in half of the included countries, which might be due to the wrong perceptions that the smoke was “filtered” through water, and that the associated risks might be minimal (34). In addition, the number of cafes or bars providing waterpipe has increased significantly as waterpipe smoking is increasingly popular among young adults (35). These findings highlight that health education programs should be strengthened to help control waterpipe smoking among adolescents.

In this study, we identified several important factors associated with waterpipe smoking. Cigarette smoking was strongly associated with waterpipe smoking. However, the causal relationship between waterpipe smoking and cigarette smoking should be made with caution because of the cross-sectional design of our study. Two other cross-sectional studies also showed that cigarette smoking was a determinant of adolescent waterpipe smoking, with OR of 3.18 (95% CI = 1.89–5.34) (36), and 6.06 (95% CI = 3.12–11.74) (37). However, a systematic review and meta-analysis based on six prospective cohort studies indicated that waterpipe smoking increased the risk of later initiation of cigarette smoking among young adults, although the definitions were not strict (the definition of waterpipe smoking was based on ever used waterpipe in four studies and cigarette use was also defined based on ever used cigarette in three studies) (38). These findings suggest policymakers should integrate waterpipe smoking with existing tobacco products when implementing policies and regulations on tobacco control.

We also found that parental smoking, closest friends' smoking, secondhand smoke exposure, tobacco advertisement exposure, and not being taught the dangers of smoking were positively associated with waterpipe smoking among adolescents. One study found that smoking of mothers or close friends were significantly associated with adolescent waterpipe smoking (39). Another study found that waterpipe smoking of family members and friends were positively associated with adolescent waterpipe smoking (37). Evidence from Lebanon suggested that secondhand smoke exposure and parental smoking were positively associated with adolescent waterpipe smoking (40). In addition, students who believed that waterpipe smoking was less harmful than cigarette smoking had a higher risk of engaging in waterpipe smoking (36). These findings highlight the importance of prevention strategies and measures that focus on providing adolescents with information about the dangers of waterpipe smoking (41). To comprehensively prevent adolescents from waterpipe smoking, waterpipe advertisements and promotions also should be monitored and restricted (42), including limiting the use of fruit flavors in waterpipe and adding labels of accurate nicotine content (43). In addition, use of education and interventions to improve adolescents' self-efficacy is also an effective way for adolescents to refuse waterpipe smoking by increasing young people's knowledge and perception of the dangers of waterpipe smoking (44).

### Strengths and limitations

There are two strengths of our study. First, the same standardized questionnaire was used in all countries on national or sub-national representative data, making the estimates directly comparable across countries. Second, we assessed the frequency of waterpipe smoking (e.g., ≥1, ≥3, and ≥6 days during the past 30 days) in 73 countries, helping distinguish between regular use and experimentation. However, several limitations of our study should be noted. First, data on waterpipe smoking were self-reported, thus there might be recall bias. Second, the GYTS data did not provide information on the types and flavors of waterpipes. Further studies are needed to better describe which types and flavors of waterpipe are more commonly used by adolescents. Third, only adolescents aged 12–16 years were included in our study, thus the estimates should not be generalized to youths of other ages. Fourth, our study was based on data collected using a cross-sectional design, thus we could not determine causal relations between waterpipe smoking and related factors. Fifth, only 8 (11.0%) of 73 countries conducted the GYTS across several cities, thus the results might not be representative of the whole country. Sixth, only 73 countries were included in our study, most countries which have higher number of adolescents population (e.g., countries from South-East Asia) were not included due to unavailable GYTS data.

## Conclusion

We found that waterpipe smoking among adolescents, especially in the European and Eastern Mediterranean regions, remains high-representing an important public health issue. It is important to establish effective prevention and control strategies to curb the epidemic of adolescent waterpipe smoking.

## Data availability statement

The datasets presented in this study can be found in online repositories. The names of the repository/repositories and accession number(s) can be found in the article/Supplementary material.

## Ethics statement

Data from the GYTS and the NYTS are de-identified and do not include any data that allow participant identification. The country data sets are publicly available and have complied with a corresponding national ethical board review. Written informed consent to participate in this study was provided by the participants' legal guardian/next of kin.

## Author contributions

BX designed the study and led the writing of the paper and was the principal investigator. CM drafted the first version of the manuscript. HY did the data analysis. HY and CM accessed and verified the data. BX, CGM, and MZ critically revised the manuscript. All authors critically revised the manuscript and approved the final version of the manuscript.

## Funding

This work was supported by the Youth Team of Humanistic and Social Science of Shandong University (20820IFYT1902). The funder of the study had no role in study design, data collection, data analyses, data interpretation, or writing of the report.

## Conflict of interest

The authors declare that the research was conducted in the absence of any commercial or financial relationships that could be construed as a potential conflict of interest.

## Publisher's note

All claims expressed in this article are solely those of the authors and do not necessarily represent those of their affiliated organizations, or those of the publisher, the editors and the reviewers. Any product that may be evaluated in this article, or claim that may be made by its manufacturer, is not guaranteed or endorsed by the publisher.

## Abbreviations

CIs: confidence intervals
GYTS: Global Youth Tobacco Surveys
NYTS: National Youth Tobacco Survey
OR: Odds Ratio
WHO: World Health Organization.
