# Supplementary material for: Prevalence of waterpipe smoking and its associated factors among adolescents aged 12–16 years in 73 countries/territories
Source: Front Public Health. 2022 Nov 17;10:1052519. doi: 10.3389/fpubh.2022.1052519 (PMC9714343; doi:10.3389/fpubh.2022.1052519)
Supplement: Supplementary file 1 [file Data_Sheet_1.doc]

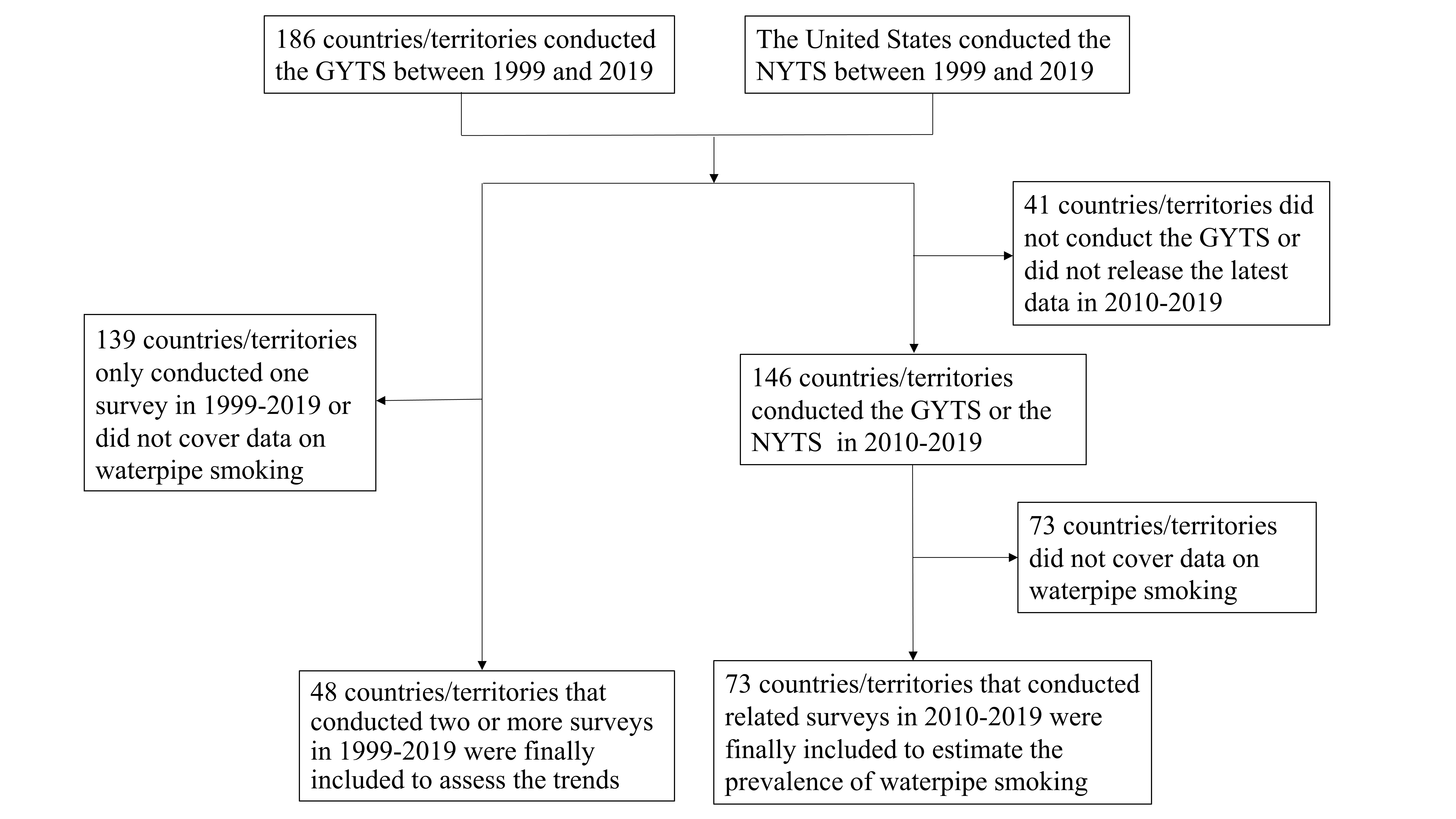


Figure S1: Flow chart of inclusion/exclusion of countries/territories

(GYTS: Global Youth Tobacco Surveys, NYTS: National Youth Tobacco Survey)

**Table S1. Characteristics of Global Youth Tobacco Surveys and U.S. National Youth Tobacco Survey of adolescents aged 12-16 years in 73 countries/territories, 2010-2019**

| **Country/territory** | **Representativeness** | **Survey year** | **Sample size** | **Boys, %** |
| --- | --- | --- | --- | --- |
| **Africa** |  |  |  |  |
| Chad | National | 2019 | 1470 | 60.7 |
| Congo | National | 2019 | 5061 | 50.4 |
| Gabon | National | 2014 | 1190 | 46.5 |
| Ghana | National | 2017 | 5481 | 50.8 |
| Kenya | National | 2013 | 1737 | 50.5 |
| Mauritania | National | 2018 | 3423 | 50.7 |
| Senegal | National | 2013 | 1234 | 54.1 |
| Seychelles | National | 2015 | 2413 | 50.1 |
| Sierra Leone | National | 2017 | 4590 | 48.4 |
| Togo | National | 2019 | 3181 | 55.2 |
| Uganda | National | 2018 | 2960 | 47.6 |
| **Americas** |  |  |  |  |
| Argentina | National | 2018 | 1405 | 49.6 |
| Bolivia | National | 2018 | 4666 | 50.2 |
| Dominican Republic | National | 2016 | 1233 | 47.4 |
| Ecuador | National | 2016 | 5094 | 50.0 |
| Guatemala | National | 2015 | 3989 | 51.8 |
| Guyana | National | 2015 | 1545 | 50.0 |
| Honduras | National | 2016 | 3334 | 47.6 |
| Jamaica | National | 2017 | 1398 | 44.8 |
| Nicaragua | National | 2019 | 7623 | 50.3 |
| Panama | National | 2017 | 2550 | 50.1 |
| Paraguay | National | 2019 | 4592 | 50.0 |
| Peru | National | 2019 | 3853 | 50.2 |
| Saint Lucia | National | 2017 | 1478 | 51.6 |
| Saint Vincent and the Grenadines | National | 2018 | 1317 | 50.1 |
| Suriname | National | 2016 | 1781 | 47.9 |
| Uruguay | National | 2014 | 4639 | 46.1 |
| United States of America | National | 2019 | 13712 | 59.2 |
| **Eastern Mediterranean** |  |  |  |  |
| Afghanistan | Subnational | 2017 | 1452 | 57.2 |
| Bahrain | National | 2015 | 3341 | 50.9 |
| Djibouti | National | 2013 | 1667 | 54.3 |
| Egypt | National | 2014 | 2303 | 52.5 |
| Gaza Strip | Subnational | 2019 | 1489 | 48.3 |
| Iraq | National | 2019 | 2076 | 55.2 |
| Jordan | National | 2014 | 2107 | 50.3 |
| Kuwait | National | 2016 | 2383 | 48.1 |
| Lebanon | National | 2011 | 2133 | 46.6 |
| Libyan Arab Jamahiriya | National | 2010 | 1761 | 48.0 |
| Morocco | National | 2016 | 3725 | 43.6 |
| Oman | National | 2016 | 2020 | 49.1 |
| Qatar | National | 2018 | 1992 | 47.5 |
| Saudi Arabia | National | 2010 | 2221 | 50.5 |
| Syrian Arab Republic | National | 2010 | 1602 | 48.8 |
| Tunisia | National | 2017 | 2402 | 50.1 |
| UNRWA GAZA (Palestine) ^a^ | Regional | 2013 | 1673 | 50.5 |
| UNRWA Jordan | Regional | 2014 | 1347 | 52.1 |
| UNRWA Lebanon | Regional | 2013 | 1408 | 45.6 |
| UNRWA West bank (Palestine) ^a^ | Regional | 2014 | 1347 | 41.2 |
| West BANK | Regional | 2016 | 1484 | 48.8 |
| Yemen | National | 2014 | 1920 | 61.2 |
| **Europe** |  |  |  |  |
| Azerbaijan | National | 2010 | 2044 | 50.0 |
| Bulgaria | National | 2015 | 4002 | 52.0 |
| Cyprus | National | 2011 | 1036 | 49.2 |
| Czech Republic | National | 2016 | 3915 | 51.4 |
| Finland | National | 2012 | 4975 | 51.0 |
| Georgia | National | 2017 | 1303 | 50.8 |
| Kyrgyzstan | National | 2019 | 6062 | 50.6 |
| Latvia | National | 2019 | 4181 | 51.4 |
| Malta | National | 2017 | 1249 | 55.7 |
| Poland | National | 2016 | 5012 | 51.2 |
| Republic of Moldova | National | 2019 | 4670 | 50.9 |
| Romania | National | 2017 | 5344 | 51.4 |
| Russian Federation | Subnational | 2015 | 6531 | 50.6 |
| Serbia | National | 2017 | 3807 | 49.6 |
| Slovakia | National | 2016 | 3965 | 50.0 |
| Slovenia | National | 2017 | 2505 | 51.5 |
| Turkey | National | 2017 | 11,3682 | 50.8 |
| Ukraine | National | 2017 | 4012 | 51.0 |
| **South-East Asia** |  |  |  |  |
| Maldives | National | 2011 | 2140 | 46.5 |
| Thailand | National | 2015 | 1864 | 51.1 |
| **Western Pacific** |  |  |  |  |
| Cambodia | National | 2016 | 2785 | 48.2 |
| Laos | National | 2016 | 5648 | 50.3 |
| Viet Nam | National | 2014 | 3528 | 49.7 |
| Total | — | — | 33,5062 | 51.1 |

^a^ Gaza and West bank are territories of Palestine.

UNRWA: United Nations Relief and Works Agency.

**Table S2. Prevalence of** **current waterpipe smoking (on ≥1 day during the past 30 days) among adolescents aged 12-16 years by frequency, sex and country/territory, 2010-2019**

| Country/territory | ≥ 1 day | | |  | ≥ 3 days | | |  | ≥ 6 days | | |
| --- | --- | --- | --- | --- | --- | --- | --- | --- | --- | --- | --- |
|  | Total | Boys | Girls |  | Total | Boys | Girls |  | Total | Boys | Girls |
| **Africa** |  |  |  |  |  |  |  |  |  |  |  |
| Chad | 0.7 (0.3-1.1) | 0.6 (0.1-1.1) | 0.9 (0.1-1.7) |  | 0.3 (0.0-0.7) | 0.3 (0.0-0.7) | 0.4 (0.0-0.8) |  | 0.1 (0.0-0.3) | 0.0 | 0.4 (0.0-0.8) |
| Congo | 2.6 (1.7-3.4) | 2.4 (1.6-3.2) | 2.7 (1.5-3.9) |  | 1.2 (0.7-1.8) | 1.1 (0.5-1.8) | 1.3 (0.5-2.1) |  | 1.0 (0.4-1.5) | 0.8 (0.3-1.4) | 1.1 (0.2-1.9) |
| Gabon | 2.4 (1.3-3.6) | 1.9 (0.0-4.3) | 2.9 (1.0-4.8) |  | 1.3 (0.9-1.7) | 0.8 (0.0-2.0) | 1.8 (0.6-2.9) |  | 1.0 (0.5-1.4) | 0.3 (0.0-0.8) | 1.5 (0.4-2.7) |
| Ghana | 5.4 (3.3-7.5) | 5.0 (3.2-6.9) | 5.8 (2.9-8.6) |  | 3.1 (1.6-4.5) | 3.1 (1.3-4.8) | 3.1 (1.2-5.0) |  | 1.7 (0.7-2.7) | 1.9 (0.5-3.4) | 1.5 (0.5-2.5) |
| Kenya | 4.8 (3.2-6.3) | 4.7 (2.7-6.7) | 4.9 (3.2-6.5) |  | 1.7 (1.0-2.4) | 1.9 (0.8-2.9) | 1.6 (0.6-2.6) |  | 1.0 (0.3-1.7) | 1.0 (0.1-1.9) | 1.0 (0.1-2.0) |
| Mauritania | 17.3 (12.0-22.5) | 16.2 (12.4-20.1) | 18.3 (10.7-25.8) |  | 9.7 (6.0-13.4) | 8.0 (5.4-10.7) | 11.3 (5.7-17.0) |  | 6.8 (3.7-10.0) | 5.4 (2.9-7.9) | 8.3 (3.8-12.8) |
| Senegal | 4.3 (2.4-6.3) | 5.3 (2.4-8.2) | 3.2 (1.2-5.2) |  | 2.2 (0.8-3.5) | 2.8 (0.8-4.9) | 1.4 (0.5-2.2) |  | 0.9 (0.5-1.4) | 1.0 (0.2-1.8) | 0.8 (0.1-1.6) |
| Seychelles | 13.5 (11.6-15.5) | 17.2 (14.5-19.8) | 9.9 (7.8-12.1) |  | 4.0 (3.1-5.0) | 5.6 (4.0-7.3) | 2.5 (1.5-3.4) |  | 2.6 (1.9-3.2) | 3.5 (2.4-4.6) | 1.7 (1.0-2.4) |
| Sierra Leone | 6.7 (3.9-9.4) | 8.5 (5.0-11.9) | 5.1 (2.6-7.5) |  | 4.1 (1.6-6.6) | 5.0 (2.1-7.9) | 3.2 (0.8-5.5) |  | 2.7 (0.8-4.7) | 3.2 (1.0-5.4) | 2.3 (0.4-4.2) |
| Togo | 1.4 (0.9-1.9) | 2.3 (1.3-3.2) | 0.4 (0.0-0.7) |  | 0.4 (0.1-0.7) | 0.7 (0.2-1.3) | 0.1 (0.0-0.2) |  | 0.3 (0.0-0.5) | 0.5 (0.0-1.0) | 0.0 |
| Uganda | 1.5 (0.7-2.3) | 1.9 (0.6-3.2) | 1.2 (0.3-2.0) |  | 0.5 (0.1-0.9) | 0.3 (0.0-0.7) | 0.7 (0.1-1.3) |  | 0.3 (0.0-0.6) | 0.2 (0.0-0.6) | 0.3 (0.0-0.8) |
| **Americas** |  |  |  |  |  |  |  |  |  |  |  |
| Argentina | 1.9 (0.8-3.0) | 2.4 (0.6-4.2) | 1.4 (0.4-2.4) |  | 0.8 (0.0-1.6) | 1.1 (0.0-2.4) | 0.5 (0.0-1.2) |  | 0.2 (0.0-0.4) | 0.3 (0.0-0.6) | 0.2 (0.0-0.4) |
| Bolivia | 2.3 (1.4-3.3) | 2.8 (1.9-3.6) | 1.9 (0.4-3.5) |  | 1.1 (0.7-1.6) | 1.3 (0.7-1.8) | 1.0 (0.3-1.7) |  | 0.7 (0.4-1.0) | 0.9 (0.4-1.3) | 0.5 (0.2-0.8) |
| Dominican Republic | 15.1 (12.7-17.5) | 15.4 (12.0-18.8) | 14.9 (12.0-17.7) |  | 5.6 (3.5-7.7) | 6.1 (3.1-9.0) | 5.2 (3.3-7.1) |  | 3.3 (1.2-5.5) | 3.5 (0.6-6.4) | 3.1 (1.4-4.9) |
| Ecuador | 4.8 (3.7-5.9) | 6.2 (4.8-7.6) | 3.5 (2.5-4.5) |  | 1.7 (1.0-2.4) | 2.3 (1.3-3.2) | 1.1 (0.7-1.5) |  | 1.2 (0.7-1.7) | 1.6 (0.9-2.4) | 0.8 (0.4-1.1) |
| Guatemala | 5.6 (4.2-6.9) | 6.6 (4.8-8.3) | 4.5 (3.2-5.7) |  | 1.2 (0.8-1.7) | 1.8 (0.9-2.6) | 0.6 (0.4-0.9) |  | 0.7 (0.4-1.0) | 1.0 (0.4-1.7) | 0.4 (0.1-0.6) |
| Guyana | 7.1 (3.8-10.5) | 9.4 (3.6-15.1) | 4.9 (2.7-7.1) |  | 4.1 (1.4-6.8) | 5.5 (0.5-10.4) | 2.7 (1.3-4.1) |  | 2.5 (0.5-4.4) | 3.1 (0.0-6.7) | 1.8 (0.8-2.8) |
| Honduras | 3.8 (3.0-4.5) | 4.0 (2.8-5.2) | 3.5 (2.7-4.3) |  | 0.4 (0.2-0.6) | 0.7 (0.3-1.2) | 0.1 (0.0-0.2) |  | 0.2 (0.1-0.4) | 0.5 (0.1-0.8) | 0.0 |
| Jamaica | 7.7 (6.3-9.1) | 11.0 (8.5-13.5) | 5.0 (3.4-6.5) |  | 4.1 (3.0-5.1) | 5.9 (4.0-7.8) | 2.6 (1.5-3.8) |  | 2.2 (1.4-3.0) | 3.3 (1.9-4.7) | 1.3 (0.5-2.1) |
| Nicaragua | 5.9 (5.0-6.8) | 7.0 (5.9-8.1) | 4.8 (3.6-5.9) |  | 1.4 (1.0-1.7) | 1.6 (1.1-2.1) | 1.2 (0.8-1.6) |  | 1.4 (1.0-1.7) | 1.6 (1.1-2.1) | 1.2 (0.8-1.6) |
| Panama | 4.0 (2.8-5.2) | 4.6 (2.9-6.3) | 3.5 (2.2-4.7) |  | 1.3 (0.8-1.9) | 1.5 (0.8-2.2) | 1.2 (0.4-2.0) |  | 0.7 (0.3-1.0) | 0.8 (0.3-1.3) | 0.5 (0.0-1.0) |
| Paraguay | 2.0 (0.8-3.1) | 2.1 (0.9-3.3) | 1.8 (0.6-3.1) |  | 0.8 (0.2-1.4) | 0.9 (0.1-1.7) | 0.7 (0.2-1.3) |  | 0.6 (0.1-1.1) | 0.8 (0.0-1.6) | 0.4 (0.0-0.9) |
| Peru | 0.7 (0.5-0.9) | 0.8 (0.4-1.2) | 0.6 (0.2-1.1) |  | 0.3 (0.2-0.5) | 0.3 (0.0-0.8) | 0.3 (0.0-0.7) |  | 0.2 (0.1-0.4) | 0.3 (0.0-0.7) | 0.2 (0.0-0.4) |
| Saint Lucia | 7.7 (5.5-10.0) | 11.2 (7.6-14.8) | 4.1 (2.5-5.8) |  | 3.0 (2.0-4.0) | 4.4 (2.5-6.3) | 1.5 (0.5-2.5) |  | 2.0 (1.1-2.9) | 3.1 (1.5-4.7) | 0.8 (0.2-1.4) |
| Saint Vincent and the Grenadines | 1.5 (0.8-2.2) | 2.0 (0.7-3.2) | 1.1 (0.4-1.7) |  | 0.7 (0.2-1.2) | 0.7 (0.0-1.5) | 0.7 (0.2-1.2) |  | 0.3 (0.0-0.5) | 0.0 | 0.5 (0.1-1.0) |
| Suriname | 8.0 (5.6-10.4) | 11.0 (7.7-14.4) | 5.3 (3.6-6.9) |  | 3.0 (1.6-4.3) | 4.5 (2.4-6.5) | 1.6 (0.4-2.7) |  | 1.6 (0.8-2.4) | 2.2 (0.8-3.7) | 1.0 (0.4-1.7) |
| United States of America | 2.2 (1.7-2.7) | 2.1 (1.5-2.7) | 2.3 (1.8-2.9) |  | 1.1 (0.8-1.4) | 1.2 (0.7-1.6) | 1.0 (0.7-1.4) |  | 0.7 (0.5-0.9) | 0.8 (0.5-1.2) | 0.5 (0.3-0.7) |
| Uruguay | 3.1 (2.4-3.9) | 3.8 (2.7-4.8) | 2.6 (1.8-3.5) |  | 0.8 (0.5-1.2) | 0.9 (0.4-1.5) | 0.7 (0.3-1.2) |  | 0.5 (0.2-0.7) | 0.7 (0.2-1.2) | 0.3 (0.1-0.5) |
| **Eastern Mediterranean** |  |  |  |  |  |  |  |  |  |  |  |
| Afghanistan | 6.2 (3.8-8.6) | 8.3 (4.7-11.8) | 3.5 (2.5-4.4) |  | 2.9 (1.5-4.3) | 3.9 (1.8-6.1) | 1.6 (0.7-2.4) |  | 1.7 (0.7-2.7) | 2.5 (0.9-4.1) | 0.7 (0.1-1.2) |
| Bahrain | 18.4 (13.1-23.6) | 26.0 (19.9-32.1) | 10.7 (8.6-12.9) |  | 10.2 (6.8-13.6) | 15.2 (11.6-18.7) | 5.3 (3.6-7.0) |  | 6.1 (4.1-8.1) | 8.8 (7.0-10.6) | 3.4 (2.2-4.5) |
| Djibouti | 12.1 (9.0-15.1) | 10.5 (7.7-13.2) | 14.0 (9.6-18.3) |  | 5.0 (3.5-6.4) | 5.0 (3.2-6.8) | 4.9 (3.1-6.7) |  | 2.8 (1.8-3.8) | 3.1 (1.7-4.4) | 2.5 (1.4-3.6) |
| Egypt | 5.8 (3.4-8.2) | 7.9 (4.8-11.1) | 3.5 (1.0-6.0) |  | 2.2 (1.2-3.1) | 3.5 (2.1-4.9) | 0.7 (0.1-1.4) |  | 0.8 (0.2-1.4) | 1.4 (0.3-2.4) | 0.2 (0.0-0.4) |
| Gaza Strip | 9.1 (4.4-13.8) | 15.5 (10.2-20.9) | 3.9 (2.1-5.7) |  | 5.7 (1.9-9.4) | 10.3 (5.5-15.0) | 1.9 (0.4-3.4) |  | 3.5 (1.0-6.1) | 6.4 (2.9-9.9) | 1.2 (0.2-2.1) |
| Iraq | 8.8 (6.0-11.6) | 10.7 (7.2-14.1) | 6.5 (3.5-9.5) |  | 5.0 (3.1-7.0) | 7.0 (4.9-9.2) | 2.6 (1.0-4.3) |  | 3.1 (1.9-4.3) | 4.1 (2.5-5.7) | 1.9 (0.3-3.1) |
| Jordan | 26.7 (22.1-31.2) | 34.6 (30.9-38.4) | 18.6 (15.4-21.8) |  | 13.8 (9.9-17.7) | 20.1 (17.4-22.9) | 7.3 (5.4-9.2) |  | 9.2 (5.9-12.5) | 14.6 (12.7-16.5) | 3.7 (2.1-5.3) |
| Kuwait | 15.5 (12.3-18.6) | 21.6 (18.1-25.2) | 9.7 (6.5-13.0) |  | 6.9 (5.2-8.5) | 10.6 (8.9-12.4) | 3.4 (2.1-4.7) |  | 4.1 (3.0-5.2) | 6.3 (4.9-7.7) | 2.1 (1.3-2.8) |
| Lebanon | 36.0 (31.2-40.8) | 41.3 (37.1-45.5) | 31.3 (24.8-37.9) |  | 17.3 (14.4-20.2) | 21.9 (19.4-24.4) | 13.2 (8.5-18.0) |  | 10.9 (9.3-12.4) | 15.3 (12.9-17.7) | 7.0 (3.9-10.1) |
| Libyan Arab Jamahiriya | 4.6 (3.4-5.8) | 6.4 (4.7-8.2) | 3.0 (1.7-4.2) |  | 1.2 (0.7-1.7) | 1.9 (1.1-2.7) | 0.6 (0.0-1.1) |  | 0.9 (0.5-1.2) | 1.4 (0.7-2.1) | 0.3 (0.0-0.7) |
| Morocco | 4.3 (2.2-6.4) | 6.6 (3.6-9.5) | 2.5 (0.8-4.2) |  | 2.0 (0.7-3.2) | 3.1 (1.0-5.1) | 1.1 (0.2-2.0) |  | 1.5 (0.5-2.6) | 2.4 (0.6-4.2) | 0.8 (0.0-1.6) |
| Oman | 1.8 (1.0-2.6) | 2.7 (1.5-3.9) | 1.0 (0.3-1.6) |  | 1.1 (0.6-1.7) | 1.8 (1.1-2.6) | 0.5 (0.0-1.1) |  | 0.6 (0.4-0.9) | 1.2 (0.5-1.8) | 0.2 (0.0-0.5) |
| Qatar | 4.2 (3.0-5.4) | 5.8 (4.3-7.2) | 2.9 (1.4-4.5) |  | 2.5 (1.6-3.3) | 3.6 (2.5-4.6) | 1.6 (0.3-2.8) |  | 1.8 (1.0-2.5) | 2.5 (1.6-3.5) | 1.1 (0.1-2.2) |
| Saudi Arabia | 9.5 (6.9-12.0) | 12.8 (8.3-17.3) | 6.1 (3.5-8.7) |  | 3.7 (2.3-5.1) | 5.3 (2.7-7.8) | 2.2 (0.9-3.5) |  | 2.6 (1.4-3.8) | 3.9 (1.7-6.2) | 1.3 (0.2-2.4) |
| Syrian Arab Republic | 20.6 (16.9-24.3) | 26.3 (21.8-30.8) | 15.2 (10.6-19.7) |  | 6.0 (3.7-8.3) | 8.6 (5.1-12.1) | 3.5 (2.0-5.1) |  | 2.3 (1.3-3.4) | 3.3 (1.5-5.0) | 1.4 (0.5-2.4) |
| Tunisia | 7.9 (6.5-9.4) | 13.4 (10.9-15.9) | 2.5 (1.4-3.5) |  | 2.7 (1.9-3.5) | 4.7 (3.3-6.1) | 0.6 (0.1-1.2) |  | 1.7 (1.0-2.3) | 2.8 (1.8-3.9) | 0.5 (0.0-0.9) |
| UNRWA GAZA (Palestine) ^a^ | 17.0 (12.0-21.9) | 22.1 (17.4-26.8) | 11.8 (7.0-16.5) |  | 8.3 (4.9-11.7) | 11.8 (8.6-14.9) | 4.8 (1.3-8.3) |  | 4.8 (2.6-7.0) | 7.1 (4.7-9.5) | 2.4 (0.1-4.8) |
| UNRWA Jordan | 20.5 (16.9-24.1) | 27.3 (25.0-29.6) | 13.3 (10.8-15.8) |  | 11.3 (8.2-14.3) | 16.6 (15.2-18.0) | 5.6 (3.6-7.6) |  | 5.9 (4.1-7.7) | 9.1 (7.3-10.9) | 2.5 (1.7-3.3) |
| UNRWA Lebanon | 23.1 (19.3-26.9) | 26.8 (20.2-33.3) | 20.1 (15.7-24.5) |  | 13.7 (10.4-16.9) | 16.4 (10.9-22.0) | 11.4 (8.4-14.4) |  | 8.7 (6.7-10.7) | 9.8 (6.6-13.1) | 7.7 (5.2-10.3) |
| UNRWA West bank (Palestine) ^a^ | 25.2 (19.2-31.2) | 33.0 (25.6-40.4) | 19.8 (14.9-24.7) |  | 12.7 (7.9-17.6) | 19.4 (10.7-28.1) | 8.1 (5.7-10.6) |  | 7.8 (4.4-11.2) | 12.2 (5.9-18.5) | 4.8 (2.9-6.7) |
| West BANK | 19.1 (16.1-22.0) | 24.6 (20.7-28.5) | 13.8 (10.3-17.2) |  | 10.9 (8.4-13.3) | 17.0 (13.9-20.1) | 5.0 (2.9-7.2) |  | 7.0 (5.1-8.9) | 11.4 (8.6-14.2) | 2.8 (1.3-4.3) |
| Yemen | 14.3 (11.5-17.1) | 17.1 (14.6-19.6) | 9.9 (6.8-13.1) |  | 6.3 (4.6-8.0) | 8.9 (7.4-10.4) | 2.4 (0.9-3.9) |  | 3.6 (2.0-5.1) | 5.3 (3.6-7.0) | 0.9 (0.1-1.7) |
| **Europe** |  |  |  |  |  |  |  |  |  |  |  |
| Azerbaijan | 4.2 (3.0-5.4) | 7.3 (5.4-9.2) | 1.0 (0.0-2.3) |  | 2.0 (1.0-3.0) | 3.4 (1.8-4.9) | 0.6 (0.0-1.4) |  | 2.0 (1.0-3.0) | 3.4 (1.8-4.9) | 0.6 (0.0-1.4) |
| Bulgaria | 18.0 (15.1-20.8) | 18.5 (14.8-22.3) | 17.3 (14.3-20.3) |  | 10.6 (8.7-12.5) | 11.1 (8.7-13.4) | 10.1 (8.2-12.1) |  | 6.2 (5.2-7.3) | 6.6 (5.2-8.0) | 5.9 (4.5-7.2) |
| Cyprus | 13.1 (11.5-14.6) | 20.5 (19.2-21.8) | 5.9 (4.1-7.7) |  | 8.2 (8.0-8.5) | 13.5 (12.1-14.9) | 3.3 (2.3-4.2) |  | 8.2 (8.0-8.5) | 13.5 (12.1-14.9) | 3.3 (2.3-4.2) |
| Czech Republic | 8.5 (7.1-9.9) | 9.0 (7.0-11.0) | 8.0 (6.3-9.6) |  | 3.2 (2.5-3.9) | 4.1 (2.8-5.3) | 2.4 (1.9-2.8) |  | 2.1 (1.4-2.8) | 2.7 (1.6-3.7) | 1.5 (1.0-2.0) |
| Finland | 2.3 (1.8-2.9) | 3.5 (2.6-4.4) | 1.1 (0.6-1.7) |  | 0.8 (0.5-1.1) | 1.4 (0.8-2.0) | 0.2 (0.0-0.4) |  | 0.8 (0.5-1.1) | 1.4 (0.8-2.0) | 0.2 (0.0-0.4) |
| Georgia | 13.0 (10.5-15.6) | 18.4 (14.5-22.3) | 7.7 (4.9-10.4) |  | 5.7 (4.2-7.2) | 8.2 (5.7-10.7) | 3.3 (1.9-4.6) |  | 3.2 (2.1-4.3) | 4.9 (3.2-6.6) | 1.5 (0.3-2.6) |
| Kyrgyzstan | 1.8 (1.1-2.5) | 2.4 (1.5-3.3) | 1.2 (0.5-1.8) |  | 0.9 (0.5-1.4) | 1.1 (0.6-1.6) | 0.8 (0.1-1.4) |  | 0.4 (0.2-0.6) | 0.5 (0.2-0.8) | 0.3 (0.1-0.5) |
| Latvia | 3.9 (3.2-4.6) | 4.3 (3.2-5.4) | 3.4 (2.4-4.4) |  | 1.3 (0.9-1.7) | 1.9 (1.1-2.7) | 0.7 (0.3-1.1) |  | 0.8 (0.4-1.1) | 1.2 (0.6-0.9) | 0.3 (0.1-0.5) |
| Malta | 8.5 (7.0-10.1) | 8.9 (6.8-11.1) | 8.0 (6.7-9.3) |  | 4.2 (3.1-5.3) | 5.2 (3.5-6.9) | 2.9 (1.5-4.3) |  | 2.5 (1.7-3.4) | 3.7 (2.3-5.2) | 1.1 (0.2-2.0) |
| Poland | 7.7 (6.7-8.6) | 9.3 (8.0-10.7) | 5.9 (4.8-7.0) |  | 3.0 (2.2-3.8) | 3.7 (2.8-4.7) | 2.3 (1.1-3.4) |  | 3.0 (2.2-3.8) | 3.7 (2.8-4.7) | 2.3 (1.1-3.4) |
| Republic of Moldova | 6.3 (5.1-7.5) | 8.0 (6.3-9.7) | 4.6 (3.1-6.1) |  | 2.7 (1.9-3.4) | 3.7 (2.4-4.9) | 1.7 (0.7-2.6) |  | 1.8 (1.3-2.4) | 2.6 (1.7-3.4) | 1.1 (0.3-1.8) |
| Romania | 36.9 (30.3-43.5) | 37.6 (30.8-44.3) | 36.2 (29.4-43.0) |  | 32.4 (25.9-39.0) | 32.2 (25.7-38.7) | 32.6 (25.7-39.5) |  | 2.2 (1.6-2.8) | 2.6 (1.8-3.3) | 1.7 (1.0-2.4) |
| Russian Federation | 8.6 (6.9-10.3) | 9.5 (7.5-11.4) | 7.7 (5.4-9.9) |  | 4.0 (2.7-5.2) | 4.9 (3.4-6.4) | 3.0 (1.8-4.2) |  | 2.7 (1.6-3.7) | 3.5 (2.3-4.7) | 1.8 (0.8-2.8) |
| Serbia | 10.0 (9.1-11.0) | 10.4 (9.0-11.8) | 9.7 (8.3-11.0) |  | 3.6 (3.0-4.1) | 4.3 (3.4-5.2) | 2.8 (2.1-3.6) |  | 1.8 (1.4-2.3) | 2.3 (1.6-3.0) | 1.4 (0.8-1.9) |
| Slovakia | 7.3 (5.8-8.8) | 8.4 (6.7-10.0) | 6.3 (4.4-8.1) |  | 2.1 (1.7-2.5) | 2.8 (2.2-3.4) | 1.4 (0.9-1.8) |  | 0.9 (0.7-1.2) | 1.5 (1.0-2.0) | 0.4 (0.2-0.7) |
| Slovenia | 10.1 (8.8-11.3) | 12.0 (10.2-13.8) | 8.0 (5.5-10.5) |  | 3.2 (2.5-3.9) | 3.4 (1.9-4.8) | 3.0 (2.2-3.8) |  | 2.1 (1.5-2.7) | 2.3 (0.9-3.6) | 2.0 (1.3-2.7) |
| Turkey | 14.1 (12.8-15.4) | 19.9 (18.0-21.8) | 8.2 (7.3-9.1) |  | 7.0 (6.2-7.8) | 10.5 (9.3-11.6) | 3.4 (3.0-3.9) |  | 4.3 (3.8-4.8) | 6.4 (5.6-7.1) | 2.2 (1.9-2.6) |
| Ukraine | 8.2 (6.5-9.8) | 9.2 (7.4-11.1) | 7.1 (5.3-8.9) |  | 3.3 (2.5-4.1) | 3.9 (2.4-5.3) | 2.7 (1.9-3.5) |  | 1.7 (1.2-2.1) | 2.1 (1.3-3.0) | 1.2 (0.8-1.6) |
| **South-East Asia** |  |  |  |  |  |  |  |  |  |  |  |
| Maldives | 2.6 (1.7-3.4) | 3.1 (1.5-4.6) | 2.1 (1.2-3.1) |  | 1.6 (1.0-2.1) | 2.1 (0.9-3.3) | 1.1 (0.2-2.0) |  | 1.0 (0.6-1.4) | 1.1 (0.3-1.9) | 0.9 (0.0-1.7) |
| Thailand | 5.5 (3.6-7.3) | 7.9 (5.6-10.2) | 2.9 (1.0-4.8) |  | 2.1 (1.2-3.1) | 3.3 (1.5-5.0) | 1.0 (0.4-1.6) |  | 1.4 (0.8-2.0) | 2.5 (1.2-3.7) | 0.2 (0.0-0.5) |
| **Western Pacific** |  |  |  |  |  |  |  |  |  |  |  |
| Cambodia | 1.6 (1.0-2.2) | 2.1 (1.2-3.0) | 1.2 (0.4-2.0) |  | 0.7 (0.3-1.0) | 0.8 (0.3-1.3) | 0.5 (0.1-1.0) |  | 0.4 (0.1-0.7) | 0.3 (0.0-0.7) | 0.5 (0.1-0.9) |
| Laos | 3.0 (2.0-4.1) | 4.2 (2.7-5.7) | 1.8 (1.0-2.6) |  | 0.9 (0.6-1.3) | 1.1 (0.6-1.6) | 0.8 (0.5-1.1) |  | 0.4 (0.2-0.5) | 0.4 (0.2-0.6) | 0.3 (0.2-1.5) |
| Viet Nam | 1.8 (1.0-2.7) | 2.4 (1.1-3.7) | 1.3 (0.5-2.1) |  | 0.4 (0.1-0.7) | 0.6 (0.1-1.0) | 0.2 (0.0-0.4) |  | 0.2 (0.0-0.3) | 0.2 (0.0-0.4) | 0.1 (0.0-0.4) |

Data are presented as % (95% CI).

^a^ Gaza and West bank are territories of Palestine.

UNRWA: United Nations Relief and Works Agency.

**Table S3. Prevalence of ever having tried/experimented with waterpipe among adolescents aged 12-16 years by sex and country/territory, 2010-2019**

| Country/territory | Total | Boys | Girls |
| --- | --- | --- | --- |
| **Africa** |  |  |  |
| Chad | 17.0 (15.0-19.0) | 17.6 (15.1-20.1) | 16.1 (13.0-19.2) |
| Congo | 12.1 (10.3-13.9) | 12.2 (9.6-14.8) | 12.0 (9.4-14.6) |
| Gabon | 6.1 (3.8-8.4) | 7.3 (4.8-9.7) | 5.1 (2.3-7.8) |
| Ghana | 8.8 (6.5-11.1) | 9.3 (6.7-11.8) | 8.4 (5.7-11.0) |
| Kenya | 5.9 (4.4-7.4) | 6.9 (5.1-8.6) | 4.9 (3.4-6.5) |
| Mauritania | 17.3 (13.9-20.7) | 18.4 (15.0-21.7) | 16.1 (11.5-20.8) |
| Senegal | 7.6 (4.9-10.3) | 8.8 (6.1-11.6) | 6.2 (2.2-10.1) |
| Seychelles | 25.7 (22.5-28.9) | 30.3 (26.7-34.0) | 21.2 (17.6-24.8) |
| Sierra Leone | 14.9 (11.2-18.5) | 17.8 (12.6-23.1) | 12.1 (9.3-15.0) |
| Togo | 5.4 (3.9-6.9) | 7.5 (5.3-9.6) | 2.9 (1.8-4.1) |
| Uganda | 6.4 (4.5-8.4) | 7.4 (5.0-9.8) | 5.6 (3.8-7.4) |
| **Americas** |  |  |  |
| Argentina | 9.4 (7.2-11.6) | 9.1 (7.2-11.1) | 9.7 (6.2-13.1) |
| Bolivia | 9.1 (6.6-11.6) | 10.2 (7.9-12.6) | 8.0 (4.4-11.5) |
| Dominican Republic | 34.3 (29.7-38.8) | 37.5 (32.5-42.6) | 31.4 (25.4-37.4) |
| Ecuador | 17.9 (13.5-22.3) | 20.8 (15.2-26.5) | 14.9 (10.6-19.2) |
| Guatemala | 6.3 (5.0-7.7) | 6.7 (4.9-8.5) | 6.0 (4.8-7.2) |
| Guyana | 9.0 (6.3-11.6) | 11.3 (7.9-14.8) | 6.7 (4.1-9.2) |
| Honduras | 4.5 (3.8-5.2) | 5.3 (4.1-6.5) | 3.8 (2.9-4.6) |
| Jamaica | 13.6 (11.8-15.4) | 15.7 (12.7-18.6) | 12.0 (9.7-14.3) |
| Nicaragua | 7.5 (6.5-8.4) | 8.9 (7.5-10.3) | 6.0 (5.1-6.9) |
| Panama | 9.8 (8.1-11.5) | 9.6 (7.5-11.6) | 10.0 (8.0-12.1) |
| Paraguay | 10.9 (7.6-14.1) | 12.6 (9.0-16.2) | 9.2 (5.9-12.4) |
| Peru | 4.4 (3.4-5.4) | 5.3 (3.9-6.8) | 3.5 (2.4-4.5) |
| Saint Lucia | 12.8 (10.3-15.2) | 16.2 (12.7-19.7) | 9.3 (7.1-11.4) |
| Saint Vincent and the Grenadines | 9.9 (8.2-11.5) | 11.7 (8.9-14.4) | 8.1 (6.0-10.3) |
| Suriname | 21.0 (17.2-24.8) | 25.2 (20.9-29.6) | 17.1 (13.9-20.3) |
| United States of America | 5.9 (4.6-7.2) | 5.8 (3.6-8.1) | 5.9 (4.9-6.9) |
| Uruguay | 6.8 (6.1-7.6) | 8.4 (7.3-9.5) | 5.5 (4.6-6.4) |
| **Eastern Mediterranean** |  |  |  |
| Afghanistan | 18.4 (15.1-21.7) | 21.3 (17.7-24.8) | 14.7 (11.6-17.8) |
| Bahrain | 26.7 (22.9-30.5) | 31.6 (26.4-36.7) | 22.2 (18.5-25.8) |
| Djibouti | 18.0 (14.0-22.0) | 17.5 (13.6-21.4) | 18.6 (13.1-24.2) |
| Egypt | 18.2 (11.8-24.6) | 21.0 (13.3-28.8) | 15.1 (8.8-21.3) |
| Gaza Strip | 38.3 (32.4-44.2) | 47.4 (42.7-52.0) | 30.7 (26.0-35.5) |
| Iraq | 34.0 (28.9-39.1) | 39.7 (35.9-43.6) | 27.2 (20.9-33.5) |
| Jordan | 45.3 (39.8-50.8) | 53.5 (48.7-58.2) | 37.1 (32.2-42.0) |
| Kuwait | 28.3 (24.0-32.5) | 33.7 (29.0-38.4) | 23.3 (18.4-28.3) |
| Lebanon | 55.4 (50.1-60.7) | 60.2 (54.6-65.7) | 51.2 (45.0-57.5) |
| Libyan Arab Jamahiriya | 13.4 (10.8-16.1) | 16.8 (13.1-20.5) | 10.3 (7.2-13.5) |
| Morocco | 10.0 (7.4-12.6) | 13.4 (9.6-17.3) | 7.4 (5.3-9.5) |
| Oman | 12.7 (9.7-15.7) | 17.2 (12.5-21.9) | 8.6 (5.1-12.1) |
| Qatar | 24.2 (21.0-27.5) | 29.7 (25.7-33.7) | 19.6 (15.9-23.4) |
| Saudi Arabia | 16.2 (13.1-19.3) | 19.7 (15.0-24.5) | 12.6 (8.7-16.6) |
| Syrian Arab Republic | 39.1 (33.4-44.8) | 46.3 (39.0-53.6) | 32.4 (24.6-40.2) |
| Tunisia | 20.6 (18.3-22.8) | 30.6 (27.3-33.8) | 10.5 (8.5-12.5) |
| UNRWA GAZA (Palestine) ^a^ | 27.9 (22.0-33.9) | 34.2 (28.8-39.7) | 21.7 (15.2-28.2) |
| UNRWA Jordan | 28.4 (24.7-32.1) | 34.2 (30.1-38.3) | 22.6 (18.4-26.8) |
| UNRWA Lebanon | 31.1 (27.1-35.0) | 32.0 (25.3-38.7) | 30.3 (25.3-35.4) |
| UNRWA West bank (Palestine) ^a^ | 38.8 (32.6-45.0) | 44.9 (36.3-53.5) | 34.8 (28.8-10.8) |
| West BANK | 37.8 (32.9-42.7) | 42.5 (36.5-48.6) | 33.5 (26.4-40.6) |
| Yemen | 26.5 (22.1-30.9) | 30.9 (25.9-35.9) | 20.1 (14.4-25.7) |
| **Europe** |  |  |  |
| Bulgaria | 38.2 (34.0-42.5) | 38.6 (33.4-43.8) | 37.8 (33.2-42.5) |
| Czech Republic | 27.3 (24.6-30.0) | 28.8 (26.4-31.3) | 25.7 (22.0-29.4) |
| Finland | 11.3 (9.8-12.8) | 13.8 (12.1-15.5) | 8.7 (6.8-10.6) |
| Georgia | 25.4 (21.2-29.6) | 32.3 (27.4-37.2) | 18.7 (14.2-23.1) |
| Kyrgyzstan | 10.1 (8.3-11.9) | 14.0 (11.5-16.4) | 6.2 (4.7-7.8) |
| Latvia | 24.4 (22.1-26.6) | 27.6 (25.0-30.3) | 20.9 (18.2-23.6) |
| Malta | 14.5 (12.5-16.5) | 13.9 (11.2-16.5) | 15.2 (12.2-18.2) |
| Republic of Moldova | 30.4 (27.4-33.4) | 36.9 (33.9-40.0) | 23.6 (20.2-27.0) |
| Romania | 12.9 (11.2-14.7) | 14.7 (12.7-16.6) | 11.1 (9.2-13.0) |
| Russian Federation | 26.7 (22.9-30.6) | 28.6 (22.6-34.6) | 24.8 (22.1-27.5) |
| Serbia | 16.3 (15.2-17.5) | 16.6 (14.9-18.3) | 16.1 (14.4-17.7) |
| Slovakia | 27.2 (24.5-29.9) | 29.0 (25.7-32.2) | 25.4 (22.3-28.6) |
| Slovenia | 27.9 (24.8-31.1) | 29.3 (25.5-33.2) | 26.5 (23.4-29.6) |
| Turkey | 28.8 (27.1-30.5) | 36.6 (34.4-38.8) | 21.1 (19.3-22.9) |
| Ukraine | 26.1 (23.2-29.0) | 30.0 (25.8-34.3) | 22.1 (19.0-25.2) |
| **South-East Asia** |  |  |  |
| Maldives | 8.6 (7.0-10.2) | 10.3 (8.5-12.0) | 7.2 (5.3-9.2) |
| Thailand | 13.6 (9.9-17.3) | 17.4 (12.3-22.5) | 9.7 (6.3-13.1) |
| **Western Pacific** |  |  |  |
| Cambodia | 6.4 (5.3-7.5) | 6.6 (4.7-8.5) | 6.2 (5.0-7.5) |
| Laos | 7.0 (5.8-8.3) | 9.3 (7.3-11.3) | 4.8 (3.7-5.8) |
| Viet Nam | 4.8 (3.4-6.3) | 5.9 (3.8-8.1) | 3.8 (2.8-4.8) |

Data are presented as % (95% CI).

^a^ Gaza and West bank are territories of Palestine. UNRWA: United Nations Relief and Works Agency.

**Table S4. Prevalence of ever having tried/experimented with waterpipe among** **adolescents aged 12-16 years by sex, age group, WHO region, World Bank income category, cigarette use, secondhand smoke exposure, and parental smoking, 2010-2019**

| Group | No. of countries | Total | Boys | Girls |
| --- | --- | --- | --- | --- |
| **Total** | 70 | 16.0 (14.8-17.1) | 18.5 (17.0-20.0) | 13.5 (12.3-14.6) ^*^ |
| **Age** |  |  |  |  |
| 12-14 years | 70 | 14.0 (13.0-15.0) | 16.0 (14.7-17.3) | 12.1 (11.0-13.2) ^*^ |
| 15-16 years | 70 | 19.4 (17.5-21.2) | 22.4 (20.1-24.7) | 16.0 (14.1-17.9) ^*^ |
| *P* value |  | <0.0001 | <0.0001 | <0.0001 |
| **WHO region** |  |  |  |  |
| African | 11 | 7.1 (6.1-8.1) | 8.1 (6.9-9.4) | 6.0 (5.0-7.0) ^*^ |
| Americas | 17 | 11.8 (9.9-13.7) | 12.2 (10.1-14.3) | 11.3 (9.3-13.3) |
| Eastern Mediterranean | 22 | 24.7 (22.4-27.1) | 29.3 (26.2-32.5) | 20.0 (17.6-22.4) ^*^ |
| European | 15 | 23.8 (22.5-25.1) | 27.4 (25.6-29.1) | 20.1 (18.8-21.5) ^*^ |
| South-East Asia | 2 | 13.6 (10.4-16.8) | 17.3 (13.0-21.7) | 9.7 (6.5-12.8) ^*^ |
| Western Pacific | 3 | 5.7 (4.8-6.5) | 6.5 (5.3-7.8) | 4.8 (4.1-5.6) ^*^ |
| *P* value |  | <0.0001 | <0.0001 | <0.0001 |
| **World Bank income** |  |  |  |  |
| Low income | 10 | 8.3 (7.1-9.6) | 9.8 (8.3-11.4) | 6.8 (5.6-7.9) ^*^ |
| Lower-middle income | 23 | 18.5 (16.3-20.6) | 21.8 (18.9-24.6) | 15.0 (13.0-17.1) ^*^ |
| Upper-middle income | 22 | 18.2 (16.3-20.0) | 20.7 (18.3-23.1) | 15.7 (13.6-17.7) ^*^ |
| High income | 15 | 10.2 (9.2-11.2) | 11.2 (9.5-12.8) | 9.2 (8.1-10.2) ^*^ |
| *P* value |  | <0.0001 | <0.0001 | <0.0001 |
| **Cigarette smoking** |  |  |  |  |
| Yes | 70 | 42.0 (37.7-46.4) | 45.0 (40.2-49.8) | 36.7 (32.2-41.2) ^*^ |
| No | 70 | 13.1 (12.1-14.2) | 14.7 (13.4-16.1) | 11.7 (10.5-12.8) ^*^ |
| *P* value |  | <0.0001 | <0.0001 | <0.0001 |
| **Secondhand smoke exposure ^#^** |  |  |  |  |
| Yes | 70 | 20.4 (19.1-21.7) | 23.6 (21.7-25.6) | 17.2 (15.6-18.5) ^*^ |
| No | 70 | 10.2 (8.8-11.6) | 11.8 (10.1-13.5) | 8.5 (7.1-9.9) ^*^ |
| *P* value |  | <0.0001 | <0.0001 | <0.0001 |
| **Parental smoking** |  |  |  |  |
| Both | 43 | 36.0 (32.7-39.2) | 38.6 (34.0-43.3) | 33.2 (29.3-37.0) ^*^ |
| Father only | 43 | 23.4 (21.1-25.6) | 28.4 (24.3-32.4) | 18.7 (16.6-20.9) ^*^ |
| Mother only | 43 | 36.5 (31.7-41.3) | 42.3 (34.7-50.0) | 31.1 (25.5-36.7) ^*^ |
| Neither | 43 | 13.2 (12.1-14.2) | 16.1 (14.5-17.7) | 10.2 (9.2-11.2) ^*^ |
| *P* value |  | <0.0001 | <0.0001 | <0.0001 |

Data are presented as % (95% CI).

WHO: World Health Organization.

^*^ There was a statistically significant difference between sexes.

**Table S5. Distribution of** **initiation age of waterpipe smoking among adolescent users aged 12-16 years by sex and country/territory, 2010-2019**

| Country/territory | ≤ 9 years | | |  | 10-13 years | | |  | 14-16 years | | |
| --- | --- | --- | --- | --- | --- | --- | --- | --- | --- | --- | --- |
|  | Total | Boys | Girls |  | Total | Boys | Girls |  | Total | Boys | Girls |
| **Africa** |  |  |  |  |  |  |  |  |  |  |  |
| Chad | 77.7 (70.3-85.1) | 73.7 (63.8-83.6) | 84.4 (73.8-95.1) |  | 6.6 (2.2-11.0) | 7.9 (1.8-14.0) | 4.4 (0.0-10.5) |  | 15.7 (9.2-22.2) | 18.4 (9.7-27.1) | 11.1 (1.9-20.3) |
| Congo | 65.4 (57.1-73.8) | 56.6 (49.4-63.7) | 73.9 (63.7-84.1) |  | 18.6 (13.2-23.9) | 21.4 (14.9-27.8) | 15.8 (10.1-21.6) |  | 16.0 (9.6-22.4) | 22.0 (15.3-28.8) | 10.3 (2.8-17.7) |
| Gabon | 19.8 (12.2-27.4) | 10.0 (2.6-17.5) | 30.3 (16.5-44.1) |  | 29.5 (21.1-38.1) | 33.8 (21.4-46.2) | 25.0 (14.9-35.1) |  | 50.7 (39.9-61.5) | 56.2 (41.3-71.2) | 44.7 (34.1-55.3) |
| Ghana | 55.4 (48.4-62.4) | 45.1 (35.2-55.0) | 64.2 (57.1-71.2) |  | 14.3 (9.1-19.6) | 16.0 (10.1-21.8) | 12.9 (5.4-20.5) |  | 30.3 (22.7-37.9) | 38.9 (27.5-50.4) | 22.9 (14.5-31.3) |
| Mauritania | 57.0 (48.6-65.4) | 57.7 (49.2-66.1) | 56.3 (45.6-66.9) |  | 23.5 (18.2-28.8) | 24.3 (19.2-29.4) | 22.7 (14.3-31.1) |  | 19.5 (14.1-24.9) | 18.1 (12.1-24.0) | 21.0 (14.4-27.6) |
| Senegal | 43.3 (26.9-5.0) | 33.0 (17.4-48.5) | 58.7 (39.5-78.0) |  | 32.8 (14.0-51.6) | 34.6 (17.9-51.3) | 30.1 (6.4-53.9) |  | 23.9 (14.1-33.7) | 32.4 (26.6-38.2) | 11.1 (6.1-16.1) |
| Sierra Leone | 49.2 (40.6-57.8) | 50.4 (42.9-57.8) | 47.5 (36.9-58.1) |  | 28.8 (17.2-40.4) | 26.2 (18.1-34.2) | 32.7 (15.2-50.3) |  | 22.0 (13.6-30.4) | 23.5 (15.3-31.6) | 19.8 (10.4-29.1) |
| Togo | 17.8 (8.3-27.3) | 14.5 (4.9-24.2) | 28.8 (11.4-46.2) |  | 46.4 (40.7-52.0) | 51.9 (45.3-58.6) | 27.5 (10.4-44.6) |  | 35.8 (28.0-43.7) | 33.5 (25.0-42.0) | 43.7 (31.6-55.8) |
| **Americas** |  |  |  |  |  |  |  |  |  |  |  |
| Bolivia | 37.1 (27.1-47.1) | 38.2 (28.3-48.1) | 35.2 (21.3-49.1) |  | 22.3 (18.5-26.1) | 21.8 (16.9-26.7) | 23.1 (18.1-28.1) |  | 40.6 (33.0-48.3) | 40.0 (32.1-47.9) | 41.7 (30.5-52.9) |
| Dominican Republic | 8.1 (3.9-12.4) | 12.7 (8.1-17.3) | 3.3 (0.0-7.4) |  | 16.0 (11.6-20.4) | 16.9 (12.7-21.1) | 15.1 (8.7-21.5) |  | 75.9 (69.3-82.4) | 70.4 (64.1-76.8) | 81.6 (73.5-89.8) |
| Ecuador | 11.2 (8.3-14.1) | 8.5 (6.4-10.6) | 15.1 (7.7-22.5) |  | 43.8 (39.8-47.7) | 45.2 (41.6-48.9) | 41.6 (35.8-47.3) |  | 45.1 (40.5-49.7) | 46.3 (41.6-50.9) | 43.3 (36.3-50.4) |
| Jamaica | 41.2 (34.3-48.2) | 45.5 (36.3-54.8) | 35.4 (25.0-45.7) |  | 22.7 (16.8-28.6) | 24.1 (16.2-32.0) | 20.7 (11.9-29.5) |  | 36.1 (29.3-42.8) | 30.4 (21.8-38.9) | 43.9 (33.1-54.7) |
| Panama | 48.4 (33.2-63.6) | 60.1 (44.1-76.0) | 36.1 (21.0-51.3) |  | 35.2 (24.8-45.5) | 28.7 (16.0-41.4) | 42.0 (29.6-54.4) |  | 16.4 (8.2-24.6) | 11.2 (3.6-18.8) | 21.9 (10.6-33.2) |
| Peru | 37.6 (30.9-44.4) | 32.0 (23.1-40.9) | 46.2 (37.1-55.2) |  | 29.5 (23.8-35.2) | 32.6 (25.4-39.7) | 24.7 (15.9-33.5) |  | 32.9 (26.6-39.2) | 35.4 (28.7-42.1) | 29.1 (20.4-37.8) |
| Saint Lucia | 44.2 (34.9-53.5) | 44.2 (34.3-54.2) | 44.1 (30.5-57.7) |  | 38.3 (30.4-46.1) | 35.6 (26.8-44.4) | 44.5 (30.6-58.4) |  | 17.5 (9.4-25.7) | 20.1 (10.2-30.1) | 11.4 (3.3-19.5) |
| Suriname | 8.1 (3.6-12.6) | 7.6 (3.1-12.1) | 8.8 (3.2-14.5) |  | 46.1 (36.4-55.9) | 50.4 (38.6-62.2) | 39.6 (30.2-49.0) |  | 45.8 (37.8-54.0) | 42.0 (32.5-51.5) | 51.5 (41.4-61.7) |
| United States of America | 9.3 (6.1-12.5) | 12.3 (8.7-15.9) | 6.2 (2.2-10.1) |  | 44.4 (38.3-50.6) | 42.0 (34.3-49.7) | 47.0 (39.7-54.3) |  | 46.3 (39.7-52.8) | 45.7 (37.7-53.7) | 46.8 (38.8-54.9) |
| **Eastern Mediterranean** |  |  |  |  |  |  |  |  |  |  |  |
| Afghanistan | 47.1 (33.5-60.6) | 39.7 (24.8-54.6) | 70.5 (56.3-84.7) |  | 23.7 (17.3-30.2) | 25.3 (17.0-33.5) | 18.8 (9.2-28.3) |  | 29.2 (18.7-39.8) | 35.0 (25.6-44.5) | 10.8 (0.3-21.2) |
| Bahrain | 39.7 (32.0-47.5) | 46.7 (41.6-51.8) | 27.9 (20.5-35.2) |  | 39.9 (35.1-44.7) | 36.0 (32.4-39.6) | 46.6 (41.5-51.7) |  | 20.4 (16.5-25.3) | 17.3 (13.8-20.9) | 25.5 (17.1-34.0) |
| Djibouti | 62.7 (54.6-70.9) | 64.9 (54.3-75.5) | 60.3 (47.8-72.8) |  | 19.6 (14.6-24.6) | 16.4 (10.3-22.5) | 23.2 (14.8-31.7) |  | 17.7 (11.4-24.0) | 18.7 (11.3-26.1) | 16.5 (8.6-24.3) |
| Egypt | 53.2 (34.8-71.6) | 43.2 (31.0-55.4) | 69.3 (38.7-99.8) |  | 33.2 (20.5-46.0) | 41.1 (30.1-52.1) | 20.4 (1.3-39.6) |  | 13.6 (2.7-24.5) | 15.6 (1.8-29.5) | 10.3 (0.0-24.1) |
| Gaza Strip | 41.3 (33.0-49.5) | 43.6 (33.9-53.3) | 38.0 (31.8-44.1) |  | 36.9 (31.0-42.9) | 33.7 (26.3-41.1) | 41.6 (31.9-51.3) |  | 21.8 (13.5-30.1) | 22.8 (14.3-31.2) | 20.5 (8.2-32.8) |
| Iraq | 24.2 (18.1-30.2) | 25.9 (19.8-31.9) | 21.1 (15.4-26.8) |  | 43.9 (36.0-51.7) | 41.2 (35.2-47.1) | 48.6 (39.1-58.2) |  | 32.0 (26.9-37.1) | 33.0 (24.8-41.2) | 30.3 (24.1-36.5) |
| Jordan | 21.8 (18.2-25.3) | 24.4 (20.8-28.0) | 17.9 (13.4-22.4) |  | 57.0 (52.8-61.3) | 57.3 (52.5-62.0) | 56.7 (50.8-62.5) |  | 21.2 (16.0-26.4) | 18.3 (15.3-21.3) | 25.4 (19.6-31.3) |
| Kuwait | 30.7 (25.1-36.4) | 30.0 (22.4-37.5) | 31.8 (24.2-39.5) |  | 36.4 (32.0-40.8) | 36.5 (31.7-41.3) | 36.2 (30.3-42.1) |  | 32.9 (26.8-39.0) | 33.5 (24.9-42.2) | 32.0 (26.9-37.1) |
| Lebanon | 17.6 (15.0-20.2) | 19.7 (15.8-23.7) | 15.4 (12.1-18.6) |  | 54.7 (51.6-57.7) | 59.0 (55.6-62.3) | 50.1 (46.0-54.3) |  | 27.7 (25.2-30.2) | 21.3 (16.8-25.8) | 34.5 (30.2-38.8) |
| Libyan Arab Jamahiriya | 35.1 (25.7-44.5) | 37.8 (27.4-48.2) | 28.7 (14.4-43.0) |  | 37.4 (28.2-46.6) | 29.3 (21.6-37.1) | 56.7 (40.7-72.7) |  | 27.5 (18.5-36.5) | 32.9 (22.6-43.1) | 14.6 (6.5-22.8) |
| Morocco | 43.8 (36.0-51.6) | 39.1 (29.0-49.1) | 53.6 (41.8-65.4) |  | 25.1 (19.9-30.2) | 28.2 (21.5-34.9) | 18.5 (7.6-29.4) |  | 31.2 (23.6-38.7) | 32.7 (25.0-40.4) | 27.9 (15.7-40.1) |
| Oman | 58.1 (46.4-69.7) | 65.3 (53.6-77.1) | 42.3 (29.9-54.8) |  | 22.4 (16.1-28.6) | 16.7 (11.9-21.5) | 34.6 (23.5-45.7) |  | 19.6 (10.9-28.2) | 18.0 (7.9-28.0) | 23.1 (9.1-37.1) |
| Qatar | 44.2 (37.2-51.1) | 48.0 (42.0-54.1) | 38.6 (31.3-45.9) |  | 39.2 (33.3-45.1) | 38.3 (33.5-43.2) | 40.4 (31.6-49.2) |  | 16.6 (12.7-20.6) | 13.6 (10.7-16.6) | 21.0 (14.2-27.9) |
| Saudi Arabia | 24.4 (17.8-30.9) | 16.7 (7.3-26.1) | 38.0 (29.9-46.1) |  | 49.6 (40.3-58.9) | 51.4 (38.9-63.8) | 46.4 (33.5-59.3) |  | 26.0 (18.8-33.3) | 31.9 (21.1-42.8) | 15.6 (7.5-23.7) |
| Syrian Arab Republic | 18.3 (12.1-24.6) | 17.6 (7.3-27.9) | 19.4 (14.8-23.9) |  | 50.1 (40.0-60.2) | 47.4 (31.6-63.3) | 53.7 (42.2-65.2) |  | 31.6 (21.6-41.5) | 35.0 (19.6-50.3) | 27.0 (17.0-36.9) |
| Tunisia | 31.2 (27.0-35.4) | 26.4 (21.6-31.2) | 47.4 (38.1-56.7) |  | 42.3 (37.2-47.3) | 43.1 (37.4-48.7) | 39.5 (31.6-47.4) |  | 26.5 (23.0-30.1) | 30.5 (26.5-34.6) | 13.1 (7.5-18.8) |
| UNRWA GAZA (Palestine) ^a^ | 41.0 (35.5-46.4) | 42.4 (37.0-47.7) | 39.0 (33.3-44.6) |  | 46.8 (42.2-51.3) | 43.9 (40.5-47.4) | 50.8 (47.0-54.5) |  | 12.3 (8.4-16.2) | 13.7 (9.3-18.1) | 10.3 (5.9-14.6) |
| UNRWA Jordan | 38.0 (31.0-45.0) | 46.3 (39.3-53.2) | 22.1 (17.1-27.1) |  | 41.9 (34.7-49.3) | 37.6 (33.1-42.1) | 50.3 (38.0-62.6) |  | 20.1 (14.1-26.0) | 16.1 (9.1-23.2) | 27.6 (18.0-37.2) |
| UNRWA Lebanon | 18.3 (13.3-23.3) | 27.4 (18.8-35.9) | 9.3 (5.9-12.7) |  | 56.9 (50.6-63.3) | 55.1 (45.7-64.6) | 58.7 (51.9-65.6) |  | 24.7 (19.7-29.8) | 17.5 (12.5-22.6) | 32.0 (26.7-37.3) |
| UNRWA West bank (Palestine) ^a^ | 34.0 (27.3-40.6) | 42.7 (37.8-47.6) | 25.9 (19.7-32.0) |  | 49.4 (42.5-56.3) | 42.8 (38.2-47.4) | 55.6 (46.1-65.0) |  | 16.6 (12.8-20.4) | 14.5 (17.8-16.3) | 18.6 (11.7-25.4) |
| West BANK | 28.3 (24.5-32.1) | 34.3 (29.2-39.5) | 20.1 (14.5-25.8) |  | 47.5 (40.6-54.2) | 47.4 (40.9-53.8) | 47.6 (35.0-60.2) |  | 24.3 (16.3-32.2) | 18.3 (13.5-23.1) | 32.3 (17.2-47.4) |
| Yemen | 29.1 (22.4-35.7) | 29.9 (22.6-37.2) | 26.6 (16.0-37.1) |  | 42.1 (33.8-50.4) | 40.1 (32.4-47.8) | 47.9 (32.5-63.3) |  | 28.8 (20.3-37.4) | 30.0 (21.1-38.8) | 25.5 (15.7-35.3) |
| **Europe** |  |  |  |  |  |  |  |  |  |  |  |
| Bulgaria | 17.5 (11.5-23.5) | 21.7 (15.2-28.3) | 13.0 (7.0-19.0) |  | 37.2 (32.1-42.4) | 37.5 (30.9-44.1) | 37.0 (30.8-43.2) |  | 45.3 (38.3-52.3) | 40.8 (31.3-50.3) | 50.0 (44.0-56.0) |
| Georgia | 27.8 (23.9-31.7) | 33.7 (28.4-38.9) | 15.7 (10.2-21.3) |  | 43.8 (36.6-51.1) | 43.1 (35.6-50.6) | 45.4 (34.2-56.6) |  | 28.4 (22.4-34.3) | 23.3 (16.4-30.1) | 38.9 (29.3-48.5) |
| Kyrgyzstan | 16.2 (13.1-19.4) | 14.7 (10.9-18.4) | 19.6 (14.4-24.8) |  | 34.8 (30.4-39.1) | 36.8 (32.2-41.4) | 30.5 (24.4-36.6) |  | 49.0 (43.1-54.8) | 48.5 (42.0-55.0) | 49.9 (42.2-57.5) |
| Malta | 11.9 (7.0-16.8) | 19.1 (10.9-27.3) | 3.8 (0.0-8.0) |  | 33.3 (26.2-40.5) | 29.2 (19.8-38.7) | 38.0 (27.3-48.7) |  | 54.8 (47.2-62.3) | 51.7 (41.3-62.1) | 58.2 (47.4-69.1) |
| Republic of Moldova | 8.5 (6.6-10.4) | 8.7 (6.2-11.2) | 8.2 (5.5-10.8) |  | 39.5 (35.3-43.7) | 41.6 (37.1-11.2) | 35.8 (29.4-42.2) |  | 52.0 (47.3-56.8) | 49.7 (44.3-55.1) | 56.1 (48.8-63.3) |
| Russian Federation | 14.1 (9.5-18.7) | 13.6 (8.3-18.9) | 14.7 (8.7-20.7) |  | 38.7 (33.0-44.4) | 41.3 (31.2-51.5) | 35.7 (30.3-41.1) |  | 47.2 (41.5-52.9) | 45.1 (36.2-54.0) | 49.6 (42.2-57.0) |
| Turkey | 19.0 (17.8-20.2) | 20.5 (19.2-21.9) | 16.4 (14.6-18.2) |  | 36.2 (33.2-39.2) | 37.6 (34.8-40.5) | 33.6 (30.0-37.2) |  | 44.8 (41.1-48.5) | 41.8 (38.2-45.4) | 50.0 (45.8-54.2) |
| Ukraine | 29.1 (24.5-33.7) | 34.0 (27.9-40.1) | 22.8 (17.4-28.3) |  | 56.9 (51.3-62.5) | 51.3 (44.2-58.4) | 64.1 (56.9-71.2) |  | 14.0 (9.1-18.8) | 14.7 (9.4-19.9) | 13.1 (7.2-18.9) |
| **South-East Asia** |  |  |  |  |  |  |  |  |  |  |  |
| Maldives | 53.8 (44.1-63.5) | 46.2 (34.9-57.5) | 63.3 (48.0-78.7) |  | 24.6 (15.1-34.0) | 26.3 (16.3-36.4) | 22.4 (11.9-32.8) |  | 21.6 (13.9-29.4) | 27.5 (16.3-38.8) | 14.3 (2.5-26.0) |
| Thailand | 18.5 (13.0-24.0) | 21.1 (15.2-27.1) | 12.7 (5.1-20.2) |  | 51.9 (44.0-59.8) | 49.7 (42.0-57.5) | 56.9 (46.1-67.6) |  | 29.6 (23.4-35.8) | 29.2 (23.1-35.2) | 30.5 (22.3-38.7) |
| **Western Pacific** |  |  |  |  |  |  |  |  |  |  |  |
| Cambodia | 52.8 (39.2-66.4) | 47.8 (28.8-66.8) | 60.7 (44.4-77.0) |  | 22.4 (11.4-33.4) | 26.4 (10.9-41.9) | 16.2 (7.0-25.4) |  | 24.8 (14.5-35.0) | 25.8 (12.9-38.7) | 23.1 (12.3-33.9) |
| Laos | 44.7 (36.2-53.2) | 35.8 (27.2-44.4) | 65.9 (60.6-71.1) |  | 27.2 (20.4-34.0) | 31.3 (23.9-38.7) | 17.4 (9.8-25.0) |  | 28.1 (22.6-33.7) | 32.9 (25.7-40.2) | 16.7 (11.2-22.2) |
| Viet Nam | 44.3 (31.6-57.0) | 42.0 (30.1-53.8) | 48.8 (43.3-54.4) |  | 11.8 (5.0-18.6) | 13.5 (7.6-19.4) | 8.7 (1.2-16.1) |  | 43.8 (30.3-57.4) | 44.5 (33.6-55.5) | 42.5 (35.3-49.7) |

Data are presented as % (95% CI).

^a^ Gaza and West bank are territories of Palestine.

UNRWA: United Nations Relief and Works Agency.

**Table S6. Distribution of initiation age of waterpipe smoking among adolescents aged 12-16 years by sex, WHO region, World Bank income category, cigarette use, secondhand smoke exposure, and parental smoking, 2010-2019**

| **Group** | No. of countries | ≤ 9 years | | |  | 10-13 years | | |  | 14-16 years | | |
| --- | --- | --- | --- | --- | --- | --- | --- | --- | --- | --- | --- | --- |
|  |  | Total | Boys | Girls |  | Total | Boys | Girls |  | Total | Boys | Girls |
| **Total** | 52 | 26.9 (23.4-30.4) | 27.0 (24.5-29.4) | 26.8 (19.2-34.4) |  | 39.5 (36.4-42.6) | 40.2 (37.1-43.4) | 38.4 (33.3-43.5) |  | 34.2 (30.8-37.5) | 33.5 (30.3-36.6) | 35.2 (29.8-40.6) |
| **WHO region** |  |  |  |  |  |  |  |  |  |  |  |  |
| Africa | 8 | 45.5 (37.1-54.0) | 36.0 (26.6-45.5) | 58.0 (43.0-72.9) ^*^ |  | 28.3 (18.2-38.4) | 31.6 (21.8-41.4) | 24.0 (11.2-36.7) |  | 26.6 (21.1-32.1) | 32.9 (27.3-38.5) | 18.3 (8.4-28.2) ^*^ |
| Americas | 9 | 12.2 (9.4-15.1) | 15.6 (12.6-18.6) | 8.4 (5.4-11.5) ^*^ |  | 25.2 (20.5-29.8) | 25.8 (21.2-30.3) | 24.5 (18.6-30.4) |  | 63.2 (57.4-69.0) | 59.2 (53.7-64.7) | 67.6 (60.5-74.8) ^*^ |
| Eastern Mediterranean | 22 | 31.8 (26.4-37.2) | 30.2 (26.3-34.0) | 34.4 (22.4-46.5) |  | 43.5 (39.3-47.8) | 43.8 (39.5-48.1) | 43.1 (34.6-51.6) |  | 25.3 (21.9-28.7) | 26.8 (22.5-31.1) | 22.8 (17.6-28.0) |
| Europe | 8 | 24.0 (21.7-26.4) | 28.1 (25.1-31.0) | 18.3 (15.5-21.2) ^*^ |  | 47.1 (43.8-50.4) | 45.0 (41.3-48.7) | 50.1 (45.6-54.6) ^*^ |  | 29.1 (26.1-32.1) | 27.2 (24.1-30.4) | 31.8 (27.5-36.0) ^*^ |
| South-East Asia | 2 | 18.7 (13.0-24.3) | 21.2 (15.2-27.3) | 13.0 (4.7-21.3) |  | 51.8 (44.0-59.6) | 49.6 (42.0-57.2) | 56.6 (42.6-70.6) |  | 29.5 (23.2-35.9) | 29.2 (22.8-35.5) | 30.4 (16.9-43.8) |
| Western Pacific | 3 | 47.0 (38.4-55.6) | 42.8 (33.6-52.0) | 54.9 (44.0-65.8) ^*^ |  | 17.4 (12.5-22.4) | 20.2 (14.5-25.9) | 12.1 (6.3-18.0) |  | 36.2 (27.0-45.5) | 37.9 (28.8-47.0) | 33.1 (21.3-44.9) |
| *P* value |  | <0.0001 | <0.0001 | <0.0001 |  | <0.0001 | <0.0001 | <0.0001 |  | <0.0001 | <0.0001 | <0.0001 |
| **World Bank income** |  |  |  |  |  |  |  |  |  |  |  |  |
| Low income | 8 | 39.7 (34.9-44.6) | 38.7 (32.4-45.0) | 41.8 (35.5-48.2) |  | 37.0 (33.7-40.3) | 35.5 (31.6-39.4) | 40.0 (35.1-44.8) |  | 23.8 (19.6-28.0) | 26.4 (21.4-31.4) | 18.5 (14.1-22.9) ^*^ |
| Lower-Middle income | 20 | 35.3 (28.9-41.7) | 32.5 (27.9-37.1) | 39.9 (26.3-53.4) |  | 40.3 (35.4-45.1) | 41.1 (36.1-46.0) | 38.9 (29.5-48.4) |  | 24.6 (20.5-28.7) | 26.6 (21.5-31.6) | 21.4 (15.2-27.5) |
| Upper-Middle income | 16 | 17.4 (15.0-19.7) | 20.5 (18.0-23.0) | 12.9 (10.1-15.8) ^*^ |  | 37.4 (32.8-42.1) | 38.6 (33.7-43.4) | 35.8 (30.0-41.7) |  | 45.9 (40.6-51.2) | 42.0 (36.8-47.2) | 51.6 (44.5-58.6) ^*^ |
| High income | 8 | 20.6 (17.4-23.8) | 20.3 (15.7-24.8) | 21.0 (17.3-24.8) |  | 46.1 (41.4-50.7) | 45.1 (38.4-51.7) | 47.5 (42.0-53.0) |  | 35.2 (30.2-40.2) | 36.0 (29.3-42.8) | 34.0 (27.7-40.3) |
| *P* value |  | <0.0001 | <0.0001 | <0.0001 |  | 0.15 | 0.39 | 0.29 |  | <0.0001 | <0.0001 | <0.0001 |
| **Cigarette use** |  |  |  |  |  |  |  |  |  |  |  |  |
| Yes | 52 | 21.5 (18.7-24.2) | 22.5 (19.0-26.0) | 18.8 (14.9-22.6) |  | 46.7 (42.4-50.9) | 46.6 (41.4-51.9) | 46.8 (41.0-52.7) |  | 32.5 (28.7-36.3) | 31.6 (26.9-36.4) | 34.9 (29.0-40.8) |
| No | 52 | 27.6 (22.9-32.3) | 27.3 (24.1-30.4) | 28.1 (18.7-37.5) |  | 38.6 (34.7-42.5) | 40.1 (36.2-44.0) | 36.8 (30.8-42.7) |  | 34.3 (30.2-38.5) | 33.3 (29.6-37.1) | 35.6 (29.2-42.0) |
| *P* value |  | 0.016 | 0.048 | 0.024 |  | 0.0030 | 0.047 | 0.0066 |  | 0.49 | 0.55 | 0.86 |
| **Secondhand smoke exposure** |  |  |  |  |  |  |  |  |  |  |  |  |
| Yes | 52 | 26.6 (22.2-30.9) | 26.8 (23.8-29.8) | 26.2 (16.7-35.8) |  | 41.0 (37.4-44.5) | 41.4 (37.7-45.2) | 40.2 (34.1-46.4) |  | 33.0 (29.5-36.5) | 32.4 (28.7-36.1) | 33.9 (28.1-39.8) |
| No | 52 | 28.2 (24.9-31.5) | 27.6 (24.1-31.1) | 29.1 (22.7-35.4) |  | 34.7 (30.5-39.0) | 36.4 (32.1-40.6) | 32.1 (26.1-38.1) |  | 37.8 (32.8-42.8) | 36.7 (32.0-41.4) | 39.5 (31.5-47.5) |
| *P* value |  | 0.54 | 0.73 | 0.62 |  | 0.015 | 0.067 | 0.053 |  | 0.050 | 0.14 | 0.18 |
| **Parental smoking** |  |  |  |  |  |  |  |  |  |  |  |  |
| Both | 30 | 30.5 (26.1-34.9) | 37.3 (30.7-43.8) | 21.6 (16.4-26.8) ^*^ |  | 43.7 (38.5-48.9) | 40.4 (32.9-48.0) | 48.1 (41.3-54.9) |  | 25.8 (20.9-30.7) | 22.3 (16.6-28.1) | 30.3 (21.8-38.8) |
| Father only | 30 | 21.2 (18.3-24.2) | 22.4 (17.9-27.0) | 19.5 (16.2-22.9) |  | 49.4 (45.3-53.6) | 46.7 (40.9-52.6) | 53.4 (48.1-58.8) |  | 29.4 (24.6-34.3) | 30.9 (23.8-38.1) | 27.2 (22.0-32.4) |
| Mother only | 30 | 22.9 (16.2-29.5) | 24.1 (14.6-33.6) | 21.2 (15.0-27.4) |  | 53.8 (42.2-65.4) | 59.9 (46.4-73.5) | 44.9 (31.4-58.3) ^*^ |  | 23.4 (15.5-31.3) | 16.0 (8.1-24.0) | 34.0 (22.3-45.7) ^*^ |
| Neither | 30 | 26.8 (23.8-29.8) | 27.0 (23.1-30.8) | 26.5 (22.8-30.2) |  | 45.7 (42.5-49.0) | 44.9 (40.4-49.3) | 47.4 (43.1-51.7) |  | 27.6 (24.8-30.3) | 28.3 (24.3-32.3) | 26.3 (22.4-30.1) |
| *P* value |  | 0.0005 | 0.0009 | 0.016 |  | 0.12 | 0.060 | 0.13 |  | 0.48 | 0.062 | 0.52 |

Data are presented as % (95% CI).

WHO: World Health Organization.

^*^ There was a statistically significant difference between sexes.

**Table S****7. Proportions of the last place to smoking waterpipe during the past 30 days among waterpipe smokers by sex and country/territory, 2010-2019**

| Country/territory | Home | |  | Coffee shop | |  | Restaurant | |  | Bar or club | |  | Other places | |
| --- | --- | --- | --- | --- | --- | --- | --- | --- | --- | --- | --- | --- | --- | --- |
|  | Boys | Girls |  | Boys | Girls |  | Boys | Girls |  | Boys | Girls |  | Boys | Girls |
| **Africa** |  |  |  |  |  |  |  |  |  |  |  |  |  |  |
| Chad | 30.8 (16.3-45.3) | 69.6 (50.7-88.4) |  | 10.3 (0.7-19.8) | 4.3 (0.0-12.7) |  | 12.8 (2.3-23.3) | 17.4 (1.9-32.9) |  | 17.9 (5.9-30.0) | 4.3 (0.0-12.7) |  | 28.2 (14.0-42.4) | 4.3 (0.0-12.7) |
| Congo | 48.5 (41.6-55.5) | 52.7 (39.7-65.8) |  | 12.3 (6.0-18.6) | 22.1 (12.0-32.2) |  | 8.8 (5.4-12.3) | 9.9 (4.5-15.3) |  | 15.3 (10.4-20.2) | 6.9 (1.7-12.1) |  | 15.1 (9.3-20.9) | 8.4 (2.1-14.8) |
| Gabon | 24.5 (17.6-31.3) | 18.3 (7.6-29.1) |  | 0.0 | 3.3 (0.0-10.0) |  | 5.3 (0.0-15.6) | 18.9 (4.7-33.1) |  | 28.5 (27.1-29.9) | 33.0 (24.7-41.3) |  | 41.8 (39.8-43.8) | 26.4 (18.1-34.7) |
| Ghana | 43.9 (32.1-55.7) | 36.5 (27.0-46.0) |  | 15.7 (6.2-25.3) | 20.2 (14.2-26.2) |  | 20.2 (4.6-35.8) | 14.9 (5.7-24.1) |  | 7.7 (4.0-11.3) | 7.4 (2.9-12.0) |  | 12.5 (4.9-20.1) | 21.0 (7.8-34.2) |
| Mauritania | 38.6 (31.2-46.0) | 31.5 (22.1-40.9) |  | 24.7 (16.5-32.9) | 21.9 (14.9-28.8) |  | 19.8 (14.8-24.8) | 19.6 (15.2-24.0) |  | 7.4 (3.5-11.2) | 11.0 (5.8-16.2) |  | 9.5 (5.3-13.8) | 16.1 (11.4-20.8) |
| Senegal | 35.9 (24.8-47.0) | 24.7 (3.3-46.1) |  | 27.3 (17.4-37.3) | 34.8 (27.1-42.6) |  | 8.6 (3.6-13.6) | 10.1 (2.9-17.4) |  | 13.5 (5.4-21.6) | 4.2 (0.0-10.1) |  | 14.6 (6.1-23.1) | 26.1 (2.4-49.9) |
| Sierra Leone | 41.3 (32.5-50.2) | 41.6 (35.9-47.3) |  | 16.9 (11.0-22.8) | 17.0 (12.5-21.6) |  | 20.5 (11.0-30.0) | 16.3 (12.6-20.0) |  | 15.9 (10.1-21.7) | 13.3 (10.3-16.3) |  | 5.3 (1.9-8.7) | 11.7 (8.3-15.1) |
| **Americas** |  |  |  |  |  |  |  |  |  |  |  |  |  |  |
| Bolivia | 21.0 (13.9-28.1) | 12.9 (8.7-17.2) |  | 32.4 (24.4-40.4) | 40.0 (32.5-47.5) |  | 23.1 (14.5-31.7) | 27.9 (19.2-36.6) |  | 3.9 (1.5-6.3) | 3.9 (0.9-6.8) |  | 19.6 (11.9-27.3) | 15.3 (5.2-25.4) |
| Dominican Republic | 30.5 (20.6-40.4) | 27.2 (18.5-35.9) |  | 7.7 (1.9-13.6) | 8.1 (4.0-12.3) |  | 3.3 (0.0-6.9) | 0.0 |  | 17.9 (4.5-31.2) | 16.7 (8.3-25.0) |  | 40.6 (31.9-49.3) | 47.9 (35.7-60.2) |
| Ecuador | 30.5 (26.8-34.1) | 20.2 (16.4-23.9) |  | 7.8 (3.8-11.8) | 10.0 (5.4-14.5) |  | 3.3 (0.7-5.8) | 4.6 (2.3-6.8) |  | 11.3 (6.8-15.8) | 14.2 (9.9-18.4) |  | 47.2 (41.4-53.0) | 51.2 (44.5-57.8) |
| Guyana | 43.2 (31.1-55.3) | 36.5 (15.2-57.8) |  | 19.1 (5.7-32.5) | 10.2 (1.7-18.7) |  | 14.8 (4.5-25.0) | 23.6 (11.4-35.9) |  | 7.4 (0.0-17.0) | 6.2 (3.9-8.6) |  | 15.6 (7.1-24.0) | 23.4 (13.0-33.8) |
| Jamaica | 47.3 (37.2-57.5) | 46.2 (30.5-61.8) |  | 15.1 (7.8-22.3) | 17.9 (5.9-30.0) |  | 16.1 (8.6-23.6) | 12.8 (2.3-23.3) |  | 4.3 (0.2-8.4) | 5.1 (0.0-12.1) |  | 17.2 (9.5-24.9) | 17.9 (5.9-30.0) |
| Panama | 38.1 (29.7-46.4) | 41.3 (29.8-52.9) |  | 27.0 (22.1-31.8) | 20.4 (11.9-28.9) |  | 12.1 (6.7-17.5) | 13.8 (8.1-19.4) |  | 3.5 (0.5-6.5) | 6.3 (0.0-13.3) |  | 19.4 (11.1-27.7) | 18.3 (11.2-25.3) |
| Paraguay | 45.8 (36.3-55.4) | 33.2 (23.7-42.6) |  | 4.0 (1.2-6.7) | 2.6 (0.0-5.7) |  | 5.8 (1.0-10.6) | 6.8 (1.6-12.0) |  | 5.6 (0.7-10.5) | 8.9 (5.4-12.5) |  | 38.8 (29.3-48.3) | 48.5 (43.0-53.9) |
| Peru | 18.3 (8.8-27.8) | 22.2 (10.2-34.2) |  | 4.5 (0.0-9.8) | 6.5 (0.0-15.6) |  | 6.9 (0.0-16.0) | 6.6 (0.4-12.7) |  | 7.8 (2.1-13.5) | 16.6 (3.1-30.0) |  | 62.5 (49.6-75.5) | 48.2 (25.3-71.0) |
| Suriname | 31.0 (21.1-41.0) | 36.0 (22.7-49.2) |  | 9.6 (3.4-15.7) | 2.7 (0.0-6.7) |  | 3.0 (0.0-6.5) | 1.3 (0.0-3.8) |  | 17.7 (9.2-26.3) | 23.7 (11.5-35.9) |  | 38.7 (28.1-49.3) | 36.4 (25.8-47.0) |
| **Eastern Mediterranean** |  |  |  |  |  |  |  |  |  |  |  |  |  |  |
| Afghanistan | 44.4 (28.6-60.2) | 44.8 (27.6-62.0) |  | 32.0 (21.0-43.1) | 36.7 (22.5-50.9) |  | 11.1 (2.6-19.7) | 3.5 (0.0-10.7) |  | 0.0 | 0.0 |  | 12.4 (6.4-18.4) | 15.0 (3.9-26.0) |
| Bahrain | 39.0 (35.9-52.2) | 39.6 (36.6-42.7) |  | 28.4 (24.5-32.3) | 26.7 (21.3-32.2) |  | 11.3 (7.7-14.9) | 12.2 (6.7-17.8) |  | 6.7 (5.0-8.4) | 4.1 (2.1-6.0) |  | 14.6 (12.0-17.1) | 17.4 (15.0-19.8) |
| Djibouti | 44.0 (37.4-50.7) | 41.9 (33.7-50.0) |  | 27.7 (19.6-35.9) | 30.5 (24.1-36.8) |  | 9.5 (4.1-14.9) | 16.0 (9.9-22.2) |  | 6.6 (2.7-10.5) | 3.7 (0.4-6.9) |  | 12.1 (7.9-16.4) | 8.0 (3.6-12.3) |
| Egypt | 29.5 (15.3-43.8) | 26.5 (5.8-47.3) |  | 42.0 (30.0-54.1) | 20.4 (5.7-35.1) |  | 14.8 (3.9-25.7) | 42.0 (15.4-68.6) |  | 0.0 | 6.3 (0.0-16.5) |  | 13.6 (2.6-24.6) | 4.8 (0.0-11.1) |
| Gaza Strip | 34.8 (30.3-39.3) | 59.1 (54.4-63.7) |  | 29.1 (24.3-33.9) | 7.8 (2.4-13.2) |  | 8.1 (3.9-12.3) | 15.2 (7.8-22.5) |  | 1.3 (0.5-2.1) | 2.9 (0.0-6.0) |  | 26.7 (20.4-33.1) | 15.1 (10.2-20.0) |
| Iraq | 39.1 (35.5-42.6) | 67.1 (59.1-75.2) |  | 33.1 (27.5-38.8) | 11.3 (5.6-17.1) |  | 8.1 (5.0-11.3) | 9.2 (4.5-13.8) |  | 3.8 (1.1-6.5) | 1.8 (0.0-3.6) |  | 15.9 (11.8-20.0) | 10.6 (7.2-13.9) |
| Jordan | 32.1 (29.2-35.0) | 69.7 (62.0-77.3) |  | 22.1 (18.3-25.9) | 9.3 (3.6-14.9) |  | 7.2 (6.1-8.4) | 2.4 (0.3-4.6) |  | 7.7 (5.4-10.1) | 1.6 (0.2-3.1) |  | 30.8 (27.4-34.3) | 17.0 (13.6-20.4) |
| Kuwait | 28.2 (20.1-36.3) | 38.1 (31.5-44.8) |  | 47.0 (36.5-57.4) | 25.3 (16.4-34.3) |  | 4.9 (2.1-7.8) | 12.5 (7.2-17.7) |  | 4.7 (0.7-8.8) | 1.1 (0.8-1.4) |  | 15.2 (11.2-19.2) | 23.0 (17.9-28.1) |
| Lebanon | 45.2 (40.2-50.1) | 56.3 (52.6-60.0) |  | 15.4 (11.6-19.3) | 4.6 (2.0-7.1) |  | 3.5 (1.7-5.4) | 7.4 (4.0-10.9) |  | 0.0 | 0.0 |  | 35.9 (32.2-39.5) | 31.7 (27.0-36.4) |
| Libyan Arab Jamahiriya | 25.8 (16.8-34.8) | 47.7 (34.3-61.1) |  | 38.4 (27.7-49.2) | 11.9 (3.1-20.8) |  | 3.6 (0.0-8.0) | 11.2 (3.1-19.2) |  | 6.6 (1.3-11.9) | 0.0 |  | 25.6 (15.6-35.6) | 29.2 (19.4-39.0) |
| Oman | 50.4 (38.0-62.9) | 38.7 (18.9-58.5) |  | 14.4 (9.0-19.9) | 18.1 (2.2-34.0) |  | 10.4 (4.7-16.0) | 22.6 (15.9-29.2) |  | 12.0 (3.5-20.5) | 0.0 |  | 12.7 (5.6-19.8) | 20.6 (8.8-32.5) |
| Qatar | 42.2 (38.1-46.4) | 47.6 (37.7-57.6) |  | 27.0 (22.6-31.4) | 22.2 (12.9-31.4) |  | 8.8 (7.4-10.2) | 7.8 (3.1-12.5) |  | 3.2 (0.9-5.5) | 5.5 (3.9-7.2) |  | 18.8 (14.3-23.2) | 16.9 (3.5-30.3) |
| Saudi Arabia | 15.1 (9.8-20.4) | 58.8 (48.1-69.4) |  | 15.7 (7.1-24.2) | 12.5 (1.1-23.9) |  | 1.5 (0.0-3.5) | 3.4 (0.5-6.3) |  | 7.6 (3.3-11.9) | 3.0 (0.0-6.4) |  | 60.1 (46.4-73.8) | 22.3 (13.9-30.8) |
| Syrian Arab Republic | 34.7 (26.3-43.1) | 55.0 (50.2-59.7) |  | 11.2 (7.5-14.9) | 7.1 (3.6-10.6) |  | 12.2 (5.3-19.1) | 13.3 (7.4-19.2) |  | 1.4 (0.0-2.9) | 0.5 (0.0-1.3) |  | 40.5 (30.2-50.7) | 24.1 (18.9-29.4) |
| Tunisia | 19.3 (13.5-25.1) | 41.1 (25.5-56.7) |  | 58.3 (51.8-64.9) | 23.7 (11.1-36.4) |  | 2.1 (0.6-3.6) | 17.7 (11.9-23.5) |  | 8.8 (5.0-12.6) | 4.2 (0.0-9.6) |  | 11.4 (7.8-15.0) | 13.3 (3.8-22.7) |
| UNRWA GAZA (Palestine) ^a^ | 42.9 (38.9-46.9) | 64.9 (59.0-70.8) |  | 25.2 (18.7-31.7) | 7.3 (4.9-9.6) |  | 6.8 (4.0-9.6) | 12.5 (4.2-20.8) |  | 5.2 (3.8-6.6) | 5.0 (1.2-8.9) |  | 19.9 (15.6-24.1) | 10.3 (5.7-14.8) |
| UNRWA Jordan | 45.9 (41.4-50.4) | 57.1 (47.4-66.7) |  | 18.6 (15.3-21.9) | 17.6 (11.9-23.3) |  | 8.6 (5.1-12.0) | 6.1 (0.9-11.3) |  | 3.5 (1.2-5.8) | 0.8 (0.0-2.4) |  | 23.5 (17.1-29.9) | 18.4 (10.8-26.0) |
| UNRWA Lebanon | 39.2 (32.8-45.6) | 54.3 (48.2-60.4) |  | 25.0 (13.6-36.4) | 9.8 (5.8-13.8) |  | 7.1 (2.5-11.8) | 7.6 (0.6-14.6) |  | 4.3 (0.7-7.9) | 1.7 (0.1-3.3) |  | 24.3 (16.2-32.5) | 26.6 (18.9-34.3) |
| UNRWA West bank (Palestine) ^a^ | 37.9 (29.2-46.7) | 63.3 (55.9-70.6) |  | 23.7 (19.0-28.4) | 5.7 (1.8-9.5) |  | 8.3 (5.2-11.4) | 8.3 (4.3-12.4) |  | 4.8 (1.2-8.3) | 1.1 (0.0-2.5) |  | 25.3 (22.3-28.2) | 21.6 (14.6-28.7) |
| West BANK | 35.2 (26.4-43.9) | 56.3 (52.2-60.4) |  | 26.1 (21.6-30.6) | 8.3 (3.6-13.0) |  | 7.9 (5.5-10.4) | 14.0 (10.0-18.1) |  | 4.8 (1.8-7.9) | 2.3 (0.0-5.1) |  | 26.0 (16.9-35.2) | 19.1 (14.4-23.9) |
| Yemen | 43.9 (33.7-54.1) | 63.2 (54.6-71.7) |  | 19.6 (11.8-27.5) | 10.4 (3.7-17.1) |  | 12.3 (8.5-16.1) | 10.3 (3.8-16.7) |  | 6.2 (2.4-9.9) | 1.8 (0.0-3.9) |  | 18.0 (9.6-26.4) | 14.4 (5.0-23.8) |
| **Europe** |  |  |  |  |  |  |  |  |  |  |  |  |  |  |
| Bulgaria | 36.1 (29.0-43.2) | 32.5 (23.2-41.7) |  | 26.7 (18.9-34.5) | 36.0 (29.4-42.6) |  | 4.8 (1.9-7.8) | 3.6 (1.8-5.4) |  | 21.4 (11.2-31.6) | 19.1 (14.2-24.0) |  | 11.0 (7.8-14.2) | 8.9 (4.8-13.0) |
| Georgia | 32.3 (19.7-45.0) | 40.1 (18.5-61.7) |  | 28.5 (16.4-40.5) | 27.4 (12.6-42.1) |  | 6.8 (3.3-10.4) | 7.6 (1.0-14.3) |  | 14.7 (7.6-21.8) | 11.1 (6.9-15.2) |  | 17.6 (10.0-25.2) | 13.8 (5.7-21.9) |
| Kyrgyzstan | 12.9 (10.4-15.5) | 24.7 (19.1-30.2) |  | 35.2 (28.6-41.8) | 34.8 (24.3-45.4) |  | 4.1 (1.3-6.9) | 8.4 (5.3-11.5) |  | 16.0 (10.8-21.2) | 9.3 (6.4-12.2) |  | 31.8 (25.9-37.7) | 22.8 (14.6-30.9) |
| Malta | 13.2 (4.1-22.3) | 11.1 (1.9-20.3) |  | 20.8 (9.8-31.7) | 2.2 (0.0-6.5) |  | 13.2 (4.1-22.3) | 6.7 (0.0-14.0) |  | 52.8 (39.4-66.3) | 80.0 (68.3-91.7) |  | 0.0 | 0.0 |
| Serbia | 19.9 (14.6-25.2) | 9.8 (5.6-14.0) |  | 57.5 (50.9-64.0) | 73.2 (67.0-79.4) |  | 0.9 (0.0-2.2) | 0.5 (0.0-1.5) |  | 9.0 (5.3-12.8) | 7.7 (4.0-11.5) |  | 12.7 (8.3-17.1) | 8.8 (4.8-12.7) |
| Slovenia | 40.5 (28.0-52.9) | 32.4 (22.9-41.8) |  | 0.0 | 0.0 |  | 0.8 (0.0-2.0) | 3.0 (0.2-5.8) |  | 34.2 (24.3-44.1) | 42.2 (33.9-50.6) |  | 24.6 (14.9-34.2) | 22.4 (15.0-29.8) |
| Ukraine | 24.6 (18.4-30.8) | 21.0 (14.2-27.8) |  | 24.8 (14.9-34.7) | 17.5 (9.2-25.7) |  | 10.3 (6.9-13.7) | 20.1 (10.4-29.8) |  | 14.4 (8.6-20.2) | 15.7 (8.5-22.8) |  | 25.9 (16.0-35.7) | 25.8 (17.4-34.1) |
| **South-East Asia** |  |  |  |  |  |  |  |  |  |  |  |  |  |  |
| Thailand | 41.7 (27.8-55.7) | 38.3 (26.8-49.9) |  | 5.0 (1.8-8.2) | 0.0 |  | 5.9 (0.8-11.0) | 1.9 (1.5-2.3) |  | 10.5 (2.4-18.6) | 25.8 (9.8-41.7) |  | 36.9 (26.9-46.8) | 34.0 (24.8-43.2) |
| **Western Pacific** |  |  |  |  |  |  |  |  |  |  |  |  |  |  |
| Cambodia | 34.8 (14.9-54.7) | 28.9 (20.2-37.7) |  | 16.7 (6.9-26.5) | 16.5 (11.9-21.1) |  | 14.2 (5.8-22.5) | 40.5 (30.2-50.9) |  | 10.6 (0.8-20.4) | 9.8 (2.2-17.5) |  | 23.8 (0.0-48.4) | 4.2 (0.0-8.6) |
| Laos | 34.7 (26.3-43.1) | 41.6 (33.1-50.1) |  | 13.6 (7.8-19.4) | 14.6 (8.3-20.8) |  | 16.4 (7.3-25.5) | 25.2 (15.4-35.0) |  | 12.5 (7.0-17.9) | 9.5 (6.4-12.5) |  | 22.9 (16.4-29.4) | 9.1 (3.7-14.4) |
| Viet Nam | 6.4 (1.1-11.8) | 3.1 (0.0-9.5) |  | 32.7 (15.0-50.3) | 26.1 (14.6-37.6) |  | 16.3 (9.9-22.8) | 9.3 (8.1-10.6) |  | 16.0 (9.9-22.2) | 10.9 (6.8-15.1) |  | 28.5 (18.9-38.2) | 50.5 (36.4-64.6) |

Data are presented as % (95% CI).

^a^ Gaza and West bank are territories of Palestine.

UNRWA: United Nations Relief and Works Agency.

**Table S8. Last places to smoking waterpipe during the past 30 days among waterpipe smokers by sex, age group, WHO region, World Bank income category, cigarette use, secondhand smoke exposure, and parental smoking, 2010-2019**

| **Group** | No. of countries | Home | |  | Coffee shop | |  | Restaurant | |  | Bar or club | |  | Other places | |
| --- | --- | --- | --- | --- | --- | --- | --- | --- | --- | --- | --- | --- | --- | --- | --- |
|  |  | Boys | Girls |  | Boys | Girls |  | Boys | Girls |  | Boys | Girls |  | Boys | Girls |
| **Total** | 48 | 33.9 (31.2-36.7) | 43.9 (40.1-47.8) ^*^ |  | 21.2 (19.0-23.5) | 12.2 (10.2-14.1) ^*^ |  | 9.0 (7.1-10.8) | 11.3 (7.5-11.0) |  | 6.8 (5.2-8.4) | 7.2 (5.2-9.2) |  | 29.1 (26.0-32.1) | 25.4 (21.8-29.0) |
| **Age group** |  |  |  |  |  |  |  |  |  |  |  |  |  |  |  |
| 12-14 years | 48 | 36.5 (32.3-40.6) | 47.3 (42.8-51.8) ^*^ |  | 20.2 (16.4-24.0) | 12.5 (9.8-15.1) ^*^ |  | 9.3 (6.6-11.9) | 11.4 (6.9-15.9) |  | 4.6 (3.4-5.8) | 5.2 (3.3-7.1) |  | 29.4 (25.0-33.8) | 23.7 (20.9-26.4) ^*^ |
| 15-16 years | 48 | 31.2 (27.7-34.8) | 39.6 (33.9-45.3) ^*^ |  | 22.3 (18.8-25.8) | 11.8 (9.2-14.5) ^*^ |  | 8.6 (5.8-11.5) | 11.1 (6.9-15.4) |  | 9.2 (6.3-12.0) | 9.8 (5.8-13.8) |  | 28.7 (25.2-32.2) | 27.6 (21.0-34.2) |
| *P* value |  | 0.058 | 0.026 |  | 0.48 | 0.73 |  | 0.76 | 0.92 |  | 0.0004 | 0.025 |  | 0.78 | 0.21 |
| **WHO region** |  |  |  |  |  |  |  |  |  |  |  |  |  |  |  |
| Africa | 7 | 38.8 (31.1-46.5) | 31.2 (17.9-44.4) |  | 22.4 (14.7-30.1) | 26.2 (19.1-33.4) |  | 13.1 (6.6-19.6) | 13.2 (8.1-18.4) |  | 12.0 (7.4-16.6) | 7.4 (3.1-11.7) |  | 13.8 (7.3-20.3) | 22.0 (9.2-34.8) ^*^ |
| Americas | 9 | 29.4 (22.8-36.1) | 26.2 (19.6-32.8) |  | 10.1 (6.0-14.1) | 10.2 (6.7-13.7) |  | 5.7 (3.0-8.4) | 2.9 (1.3-4.5) ^*^ |  | 14.4 (5.4-23.4) | 15.4 (8.8-22.0) |  | 40.4 (34.2-46.6) | 45.3 (35.3-55.3) |
| Eastern Mediterranean | 21 | 34.1 (30.7-37.6) | 54.4 (49.4-59.5) ^*^ |  | 25.1 (22.0-28.2) | 11.0 (8.6-13.5) ^*^ |  | 9.6 (7.0-12.2) | 14.3 (8.5-20.0) |  | 3.4 (2.7-4.2) | 2.0 (0.7-3.3) |  | 27.8 (23.6-31.9) | 18.3 (16.1-20.5) ^*^ |
| Europe | 7 | 28.8 (23.5-34.1) | 28.2 (21.3-35.0) |  | 25.4 (19.4-31.3) | 22.4 (16.5-28.2) |  | 7.7 (5.7-9.8) | 13.3 (8.2-18.5) ^*^ |  | 16.5 (12.8-20.2) | 16.2 (11.9-20.4) |  | 21.6 (16.3-27.0) | 19.9 (15.2-24.7) |
| South-East Asia | 1 | 41.7 (28.3-55.2) | 38.3 (25.7-51.0) |  | 5.0 (0.5-9.6) | 0.0 |  | 5.9 (1.0-10.7) | 1.9 (0.0-5.8) |  | 10.5 (2.8-18.1) | 25.8 (10.7-40.8) ^*^ |  | 36.9 (26.3-47.4) | 34.0 (23.6-44.4) |
| Western Pacific | 3 | 21.8 (13.4-30.3) | 19.1 (11.4-26.8) |  | 23.6 (12.9-34.4) | 20.5 (11.6-29.4) |  | 15.4 (10.6-20.2) | 24.8 (16.9-32.7) ^*^ |  | 13.3 (6.2-20.4) | 10.3 (2.3-18.2) |  | 25.8 (14.4-37.3) | 25.3 (15.5-35.1) |
| *P* value |  | 0.093 | <0.0001 |  | <0.0001 | <0.0001 |  | 0.050 | <0.0001 |  | <0.0001 | <0.0001 |  | <0.0001 | <0.0001 |
| **World Bank income** |  |  |  |  |  |  |  |  |  |  |  |  |  |  |  |
| Low income | 7 | 43.4 (36.3-50.5) | 54.5 (48.3-60.6) ^*^ |  | 26.4 (20.2-32.6) | 18.1 (13.0-23.2) ^*^ |  | 10.3 (6.3-14.3) | 8.6 (5.3-11.8) |  | 3.4 (2.3-4.4) | 3.2 (2.0-4.4) |  | 16.5 (13.2-19.9) | 15.7 (11.6-19.8) |
| Lower-Middle income | 18 | 33.2 (29.0-37.4) | 43.3 (37.7-48.8) ^*^ |  | 25.0 (21.4-28.5) | 15.1 (12.0-18.2) ^*^ |  | 12.0 (8.8-15.2) | 18.6 (12.1-25.2) ^*^ |  | 4.8 (3.7-5.8) | 4.0 (2.3-5.7) |  | 25.0 (20.1-30.0) | 19.0 (15.9-22.0) ^*^ |
| Upper-Middle income | 15 | 36.0 (31.8-40.2) | 42.6 (36.3-49.0) |  | 16.1 (12.8-19.5) | 8.1 (5.7-10.4) ^*^ |  | 5.7 (4.0-7.3) | 3.6 (1.9-5.4) |  | 9.6 (5.8-13.4) | 11.6 (7.5-15.7) |  | 32.6 (28.6-36.5) | 34.1 (27.0-41.1) |
| High income | 8 | 23.4 (18.8-28.0) | 53.6 (46.1-61.1) ^*^ |  | 18.5 (12.5-24.6) | 14.3 (6.0-22.5) |  | 3.8 (2.2-5.4) | 5.8 (3.6-8.0) |  | 8.5 (5.5-11.6) | 4.6 (2.0-7.2) |  | 45.7 (35.1-56.3) | 21.7 (15.5-28.0) ^*^ |
| *P* value |  | 0.0048 | 0.17 |  | <0.0001 | 0.0004 |  | <0.0001 | <0.0001 |  | <0.0001 | <0.0001 |  | <0.0001 | <0.0001 |
| **Cigarette smoking** |  |  |  |  |  |  |  |  |  |  |  |  |  |  |  |
| Yes | 48 | 27.9 (23.0-32.7) | 35.0 (28.5-41.6) |  | 27.0 (22.1-32.0) | 14.0 (8.2-19.8) ^*^ |  | 7.7 (3.5-11.9) | 10.0 (3.4-16.6) |  | 7.6 (4.9-10.4) | 10.5 (6.0-15.0) |  | 29.8 (25.5-34.1) | 30.5 (23.9-37.0) |
| No | 48 | 36.4 (32.6-40.2) | 45.9 (41.9-49.9) ^*^ |  | 18.9 (15.7-22.0) | 11.3 (8.9-13.7) ^*^ |  | 8.4 (7.0-9.8) | 11.6 (8.2-15.0) |  | 6.4 (4.4-8.3) | 6.4 (4.1-8.8) |  | 30.0 (25.1-34.8) | 24.8 (20.4-29.3) |
| *P* value |  | 0.0090 | 0.0033 |  | 0.0062 | 0.41 |  | 0.76 | 0.56 |  | 0.34 | 0.090 |  | 0.96 | 0.18 |
| **Secondhand smoke exposure** |  |  |  |  |  |  |  |  |  |  |  |  |  |  |  |
| Yes | 48 | 32.8 (29.8-35.8) | 46.3 (42.4-50.2) ^*^ |  | 22.1 (19.5-24.7) | 12.3 (10.0-14.5) ^*^ |  | 9.3 (7.1-11.5) | 10.7 (6.7-14.7) |  | 6.9 (5.2-8.6) | 6.4 (4.6-8.2) |  | 29.0 (25.7-32.2) | 24.4 (21.0-27.8) |
| No | 48 | 38.8 (33.5-44.0) | 36.5 (29.9-43.1) |  | 17.9 (13.8-21.9) | 11.5 (7.6-15.4) ^*^ |  | 7.8 (5.7-10.0) | 13.4 (8.5-18.2) ^*^ |  | 6.7 (3.9-9.4) | 10.2 (5.4-15.0) |  | 28.9 (23.5-34.3) | 28.5 (22.2-34.7) |
| *P* value |  | 0.033 | 0.0040 |  | 0.088 | 0.73 |  | 0.34 | 0.25 |  | 0.86 | 0.055 |  | 0.98 | 0.12 |
| **Parental smoking** |  |  |  |  |  |  |  |  |  |  |  |  |  |  |  |
| Both | 28 | 44.8 (35.2-54.4) | 52.3 (45.7-58.8) |  | 15.9 (11.3-20.5) | 16.4 (11.6-21.3) |  | 8.5 (3.4-13.7) | 8.3 (3.0-13.5) |  | 4.9 (2.5-7.3) | 3.2 (1.6-4.8) |  | 25.9 (19.0-32.8) | 19.8 (14.1-25.5) |
| Father only | 28 | 34.2 (28.1-40.4) | 55.4 (50.8-60.0) ^*^ |  | 14.4 (10.9-17.9) | 8.8 (6.2-11.4) ^*^ |  | 9.9 (4.8-15.0) | 7.9 (5.6-10.2) |  | 7.0 (4.3-9.7) | 5.2 (2.3-8.1) |  | 34.5 (28.4-40.6) | 22.7 (18.5-26.8) ^*^ |
| Mother only | 28 | 28.7 (15.5-41.8) | 41.9 (30.6-53.3) ^*^ |  | 11.4 (5.2-17.7) | 12.3 (2.6-22.1) |  | 7.2 (3.1-11.3) | 20.2 (4.2-36.2) ^*^ |  | 5.4 (1.2-9.6) | 4.2 (1.4-6.9) |  | 47.4 (28.4-66.3) | 21.4 (13.4-29.4) ^*^ |
| Neither | 28 | 32.8 (28.5-37.1) | 48.0 (44.1-51.9) ^*^ |  | 19.6 (16.8-22.5) | 9.7 (7.6-11.8) ^*^ |  | 8.2 (5.7-10.6) | 12.9 (10.0-15.8) ^*^ |  | 5.5 (3.7-7.4) | 3.7 (2.1-5.3) |  | 33.9 (28.6-39.2) | 25.7 (22.2-29.2) ^*^ |
| *P* value |  | 0.12 | 0.036 |  | 0.028 | 0.024 |  | 0.84 | 0.029 |  | 0.61 | 0.44 |  | 0.10 | 0.30 |

Data are presented as % (95% CI).

WHO: World Health Organization.

^*^ There was a statistically significant difference between sexes.

**Table S9. Trends in the prevalence of waterpipe smoking (on ≥1 day during the past 30 days) among adolescents aged 12-16 years from 2000 to 2019 by country/territory**

| Country/territory | Representativeness | Survey year | First year,  %(95%CI) | Last year,  %(95%CI) | Total absolute change, % | Absolute change/5-years, % | *P for trend* |
| --- | --- | --- | --- | --- | --- | --- | --- |
| **Americas** |  |  |  |  |  |  |  |
| Argentina | National | 2012, 2018 | 6.5 (5.3-7.7) | 1.9 (0.8-3.0) | -4.6 | -3.8 | <0.0001 |
| Bolivia | National | 2012, 2018 | 9.2 (3.3-15.1) | 2.3 (1.4-3.3) | -6.9 | -5.8 | <0.0001 |
| Nicaragua | National | 2014, 2019 | 5.0 (4.0-6.0) | 5.9 (4.9-6.9) | 0.9 | 0.9 | <0.0001 |
| Paraguay | National | 2014, 2019 | 4.4 (3.3-5.5) | 2.0 (1.0-3.0) | -2.4 | -2.4 | <0.0001 |
| Peru | National | 2014, 2019 | 2.2 (0.9-3.5) | 0.7 (0.4-1.0) | -1.5 | -1.5 | <0.0001 |
| United States of America | National | 2011, 2012, 2013, 2014, 2015, 2016, 2017, 2018, 2019 | 2.0 (1.6-2.4) | 2.2 (1.7-2.7) | 0.2 | 0.1 | <0.0001 |
| **Eastern Mediterranean** |  |  |  |  |  |  |  |
| Djibouti | National | 2003, 2009, 2013 | 8.1 (6.2-10.1) | 12.1 (9.1-15.0) | 4.0 | 2.0 | <0.0001 |
| Egypt | National | 2001, 2009, 2014 | 5.6 (3.7-7.5) | 5.8 (3.7-7.9) | 0.2 | 0.1 | <0.0001 |
| Gaza Strip | Subnational | 2000, 2013, 2019 | 11.6 (8.1-15.1) | 9.1 (4.9-13.4) | -2.5 | -0.7 | <0.0001 |
| Iraq | National | 2014, 2019 | 12.2 (8.2-16.2) | 8.8 (5.7-11.9) | -3.4 | -3.4 | <0.0001 |
| Jordan | National | 2009, 2014 | 20.5 (16.9-24.1) | 26.7 (22.6-30.7) | 6.2 | 6.2 | <0.0001 |
| Kuwait | National | 2009, 2016 | 13.9 (11.7-16.0) | 15.5 (12.3-18.6) | 1.6 | 1.1 | <0.0001 |
| Lebanon | National | 2001, 2005, 2011 | 36.1 (33.2-39.1) | 36.0 (30.8-41.2) | -0.1 | -0.1 | 0.74 |
| Morocco | National | 2010, 2016 | 6.0 (3.5-8.5) | 4.3 (2.4-6.2) | -1.7 | -1.4 | <0.0001 |
| Oman | National | 2002, 2010, 2016 | 6.3 (4.1-8.6) | 1.8 (1.0-2.6) | -4.5 | -1.6 | <0.0001 |
| Qatar | National | 2007, 2013, 2018 | 10.9 (8.0-13.7) | 4.2 (2.9-5.5) | -6.7 | -3.0 | <0.0001 |
| Saudi Arabia | National | 2007, 2010 | 6.1 (4.9-7.2) | 9.5 (6.8-12.2) | 3.4 | 5.7 | <0.0001 |
| Syrian Arab Republic | National | 2007, 2010 | 24.6 (20.8-28.4) | 20.6 (16.5-24.7) | -4.0 | -6.7 | <0.0001 |
| Tunisia | National | 2007, 2010, 2017 | 7.4 (6.0-8.7) | 7.9 (6.4-9.5) | 0.5 | 0.3 | <0.0001 |
| United Arab Emirates | National | 2002, 2005 | 8.9 (6.7-11.0) | 10.1 (8.6-11.6) | 1.2 | 2.0 | <0.0001 |
| UNRWA GAZA (Palestine) ^a^ | Regional | 2008, 2013 | 13.0 (9.5-16.6) | 17.0 (12.7-21.3) | 4.0 | 4.0 | <0.0001 |
| UNRWA Jordan | Regional | 2008, 2014 | 19.9 (15.0-24.7) | 20.5 (16.5-24.5) | 0.6 | 0.5 | 0.027 |
| UNRWA Lebanon | Regional | 2008, 2013 | 35.6 (29.6-41.7) | 23.1 (19.1-27.2) | -12.5 | -12.5 | <0.0001 |
| UNRWA west bank | Regional | 2008, 2014 | 29.7 (23.8-35.7) | 25.2 (19.9-30.5) | -4.5 | -3.8 | <0.0001 |
| West BANK (Palestine) ^a^ | Regional | 2000, 2009, 2016 | 21.6 (18.9-24.4) | 19.1 (15.0-23.1) | -2.5 | -0.8 | <0.0001 |
| Yemen | National | 2008, 2014 | 3.9 (2.0-5.8) | 14.3 (11.6-16.9) | 10.4 | 8.7 | <0.0001 |
| **Europe** |  |  |  |  |  |  |  |
| Czech Republic | National | 2011, 2016 | 22.0 (18.7-25.2) | 8.5 (7.0-10.0) | -13.5 | -13.5 | <0.0001 |
| Georgia | National | 2014, 2017 | 9.3 (6.5-12.0) | 13.0 (10.0-16.1) | 3.7 | 6.2 | <0.0001 |
| Kyrgyzstan | National | 2014, 2019 | 2.4 (1.6-3.1) | 1.8 (1.1-2.5) | -0.6 | -0.6 | <0.0001 |
| Latvia | National | 2007, 2011, 2014, 2019 | 32.3 (28.0-36.6) | 3.9 (3.0-4.7) | -23.2 | -9.7 | <0.0001 |
| Poland | Subnational | 2009, 2016 | 11.0 (8.6-13.3) | 7.7 (6.6-8.7) | -3.3 | -2.4 | <0.0001 |
| Romania | National | 2009, 2013, 2017 | 2.3 (1.0-3.6) | 36.9 (30.6-43.1) | 34.6 | 21.6 | <0.0001 |
| Slovakia | National | 2007, 2011, 2016 | 4.9 (4.0-5.9) | 7.3 (5.9-8.7) | 2.4 | 1.3 | <0.0001 |
| Slovenia | National | 2011, 2017 | 7.7 (5.1-10.2) | 10.1 (8.5-11.6) | 2.4 | 2.0 | <0.0001 |
| Turkey | National | 2009, 2012, 2017 | 6.5 (5.1-7.9) | 14.1 (12.9-15.3) | 7.6 | 4.8 | <0.0001 |
| Republic of Moldova | National | 2013, 2019 | 7.7 (5.7-9.7) | 6.3 (5.3-7.3) | -1.4 | -1.2 | <0.0001 |
| Ukraine | National | 2011, 2017 | 13.6 (10.7-16.4) | 8.2 (6.2-10.2) | -5.4 | -4.5 | <0.0001 |
| **South-East Asia** |  |  |  |  |  |  |  |
| Maldives | National | 2007, 2011 | 0.1 (0.0-0.3) | 2.6 (1.7-3.5) | 2.5 | 3.1 | <0.0001 |

Data are presented as % (95% CI).

^a^ Gaza and West bank are territories of Palestine.

UNRWA: United Nations Relief and Works Agency.

**Table S10. Linear trends per 5 calendar years in the prevalence of waterpipe smoking (on ≥1 day during the past 30 days) among adolescents aged 12-16 years from 2000 to 2019**

| **Group** | No. of  countries | Total | Boys | Girls |
| --- | --- | --- | --- | --- |
| **Total** | 38 | -0.28 (-2.23 to 1.71) | -0.76 (-2.96 to 1.45) | -0.01 (-1.83 to 1.85) |
| **WHO region** |  |  |  |  |
| Americas | 6 | -2.08 (-4.68 to 0.53) | -2.24 (-4.38 to -0.10) | -1.98 (-5.19 to 1.24) |
| Eastern Mediterranean | 20 | -0.17 (-2.35 to 2.01) | -0.81 (-3.47 to 1.84) | 0.16 (-1.59 to 1.91) |
| Europe | 11 | 0.15 (-6.14 to 6.49) | -0.28 (-6.98 to 6.43) | 0.62 (-5.45 to 6.68) |
| South-East Asia | 1 | 3.13 | 3.88 | 2.38 |
| **World Bank income** |  |  |  |  |
| Low income | 4 | -2.94 (-14.27 to 8.39) | -3.81 (-16.38 to 8.76) | -2.56 (-13.06 to 7.95) |
| Lower-Middle income | 14 | -0.27 (-2.65 to 2.11) | -0.54 (-3.41 to 2.33) | 0.19 (-1.85 to 2.22) |
| Upper-Middle income | 9 | 2.79 (-3.28 to 8.86) | 2.38 (-4.34 to 9.10) | 2.68 (-2.98 to 8.35) |
| High income | 11 | -2.59 (-7.71 to 2.52) | -2.49 (-6.66 to 1.68) | -2.22 (-7.08 to 2.63) |

Data are presented as % (95% CI).

**Table S11. Factors associated with waterpipe smoking (on ≥1 day during the past 30 days) among adolescents aged 12-16 years by sex**

| Variable | Boys | | |  | Girls | | |
| --- | --- | --- | --- | --- | --- | --- | --- |
|  | Prevalence (%) | *β* | OR (95% CI) |  | Prevalence (%) | *β* | OR (95% CI) |
| **Age group** |  |  |  |  |  |  |  |
| 12-14 years | 6.9 |  | 1.00 |  | 4.8 |  | 1.00 |
| 15-16 years | 11.1 | 0.112 | 1.19 (0.96-1.31) |  | 6.1 | 0.289 | 1.34 (1.09-1.64) |
| **Parental smoking** |  |  |  |  |  |  |  |
| Neither | 7.7 |  | 1.00 |  | 4.4 |  | 1.00 |
| Father only | 14.9 | 0.408 | 1.50 (1.25-1.81) |  | 8.0 | 0.425 | 1.53 (1.32-1.78) |
| Mother only | 30.7 | 1.244 | 3.47 (2.25-5.36) |  | 17.4 | 0.891 | 2.44 (1. 86-3.20) |
| Both | 26.3 | 0.798 | 2.22 (1.80-2.74) |  | 19.6 | 0.926 | 2.52 (2.03-3.13) |
| **Smoking status of closest friends** |  |  |  |  |  |  |  |
| None | 4.8 |  | 1.00 |  | 3.7 |  | 1.00 |
| Some | 13.8 | 0.712 | 2.04 (1.71-2.42) |  | 9.9 | 0.865 | 2.37 (2.01-2.81) |
| Most | 26.7 | 1.094 | 2.99 (2.44-3.66) |  | 14.8 | 0.978 | 2.66 (2.10-3.37) |
| All | 43.1 | 1.561 | 4.77 (3.51-6.47) |  | 25.0 | 1.176 | 3.24 (2.23-4.71) |
| **Cigarette smoking** |  |  |  |  |  |  |  |
| No | 4.9 |  | 1.00 |  | 3.6 |  | 1.00 |
| Yes | 32.8 | 1.534 | 4.64 (3.85-5.56) |  | 25.3 | 1.657 | 5.24 (4.25-6.47) |
| **Secondhand smoke exposure** |  |  |  |  |  |  |  |
| No | 3.5 |  | 1.00 |  | 2.6 |  | 1.00 |
| Yes | 12.3 | 0.471 | 1.60 (1.38-1.86) |  | 7.3 | 0.446 | 1.56 (1.27-1.93) |
| **Tobacco advertisements exposure** |  |  |  |  |  |  |  |
| No | 5.3 |  | 1.00 |  | 3.5 |  | 1.00 |
| Yes | 9.6 | 0.263 | 1.30 (1.12-1.51) |  | 5.9 | 0.137 | 1.15 (0.98-1.34) |
| **Being taught about dangers of smoking** |  |  |  |  |  |  |  |
| Yes | 7.9 |  | 1.00 |  | 5.1 |  | 1.00 |
| No | 9.9 | 0.178 | 1.19 (1.06-1.35) |  | 5.9 | 0.278 | 1.32 (1.13-1.54) |
| **Survey year** |  |  |  |  |  |  |  |
| 2016-2019 | 6.5 |  | 1.00 |  | 4.6 |  | 1.00 |
| 2010-2015 | 10.7 | 0.416 | 1.52 (1.25-1.85) |  | 6.0 | 0.440 | 1.55 (1.29-1.88) |

All variables listed in the table were introduced into logistic regression models.

OR: odds ratio; CI: confidence interval.

**Table S12. Factors associated with waterpipe smoking (on ≥1 day during the past 30 days) among adolescents aged 12-16 years by World Bank income category**

| Variable | Low income | | |  | Lower-Middle income | | |  | Upper-Middle income | | |  | High income | | |
| --- | --- | --- | --- | --- | --- | --- | --- | --- | --- | --- | --- | --- | --- | --- | --- |
|  | Prevalence (%) | *β* | OR (95% CI) |  | Prevalence (%) | *β* | OR (95% CI) |  | Prevalence (%) | *β* | OR (95% CI) |  | Prevalence (%) | *β* | OR (95% CI) |
| **Sex** |  |  |  |  |  |  |  |  |  |  |  |  |  |  |  |
| Girls | 4.0 |  | 1.00 |  | 5.6 |  | 1.00 |  | 5.9 |  | 1.00 |  | 3.8 |  | 1.00 |
| Boys | 5.2 | -0.332 | 0.72 (0.43-2.32) |  | 10.5 | 0.251 | 1.29 (1.03-1.60) |  | 8.7 | 0.219 | 1.24 (1.10-1.41) |  | 5.6 | 0.482 | 1.62 (1.19-2.21) |
| **Age group** |  |  |  |  |  |  |  |  |  |  |  |  |  |  |  |
| 12-14 years | 4.8 |  | 1.00 |  | 7.0 |  | 1.00 |  | 5.8 |  | 1.00 |  | 3.4 |  | 1.00 |
| 15-16 years | 4.4 | 0.378 | 1.46 (0.92-2.32) |  | 10.0 | -0.052 | 0.95 (0.76-1.19) |  | 10.2 | -0.142 | 0.87 (0.76-0.99) |  | 6.5 | -0.185 | 0.83 (0.64-1.09) |
| **Parental smoking** |  |  |  |  |  |  |  |  |  |  |  |  |  |  |  |
| Neither | 2.8 |  | 1.00 |  | 7.2 |  | 1.00 |  | 8.4 |  | 1.00 |  | 5.8 |  | 1.00 |
| Father only | 11.8 | 0.422 | 1.53 (0.78-3.02) |  | 13.2 | 0.378 | 1.46 (1.20-1.78) |  | 9.1 | 0.059 | 1.06 (0.94-1.20) |  | 10.3 | 0.094 | 1.10 (0.84-1.43) |
| Mother only | 26.6 | 1.604 | 4.97 (1.34-18.54) |  | 23.0 | 0.859 | 2.36 (1.64-3.39) |  | 26.9 | 0.256 | 1.29 (1.01-1.66) |  | 22.2 | 1.299 | 3.66 (1.86-7.21) |
| Both | 16.9 | 0.330 | 1.39 (0.50-3.85) |  | 24.9 | 0.934 | 2.54 (1.89-3.43) |  | 28.5 | 0.273 | 1.31 (1.08-1.60) |  | 14.1 | 0.413 | 1.51 (1.13-2.03) |
| **Smoking status of closest friends** |  |  |  |  |  |  |  |  |  |  |  |  |  |  |  |
| None | 2.3 |  | 1.00 |  | 5.7 |  | 1.00 |  | 4.7 |  | 1.00 |  | 2.9 |  | 1.00 |
| Some | 11.4 | 1.314 | 3.72 (2.15-6.45) |  | 17.0 | 0.681 | 1.98 (1.58-2.47) |  | 7.7 | 0.427 | 1.53 (1.33-1.77) |  | 13.6 | 0.896 | 2.45 (1.68-3.58) |
| Most | 26.9 | 1.443 | 4.23 (2.09-8.56) |  | 26.6 | 1.093 | 2.98 (2.25-3.94) |  | 15.5 | 0.719 | 2.05 (1.64-2.56) |  | 21.2 | 1.223 | 3.40 (2.47-4.68) |
| All | 41.3 | 2.142 | 8.52 (3.69-19.6) |  | 40.2 | 1.548 | 4.70 (2.88-7.68) |  | 34.2 | 1.000 | 2.72 (1.90-3.90) |  | 30.0 | 1.227 | 3.41 (2.13-5.46) |
| **Cigarette smoking** |  |  |  |  |  |  |  |  |  |  |  |  |  |  |  |
| No | 2.4 |  | 1.00 |  | 5.0 |  | 1.00 |  | 4.8 |  | 1.00 |  | 2.5 |  | 1.00 |
| Yes | 35.3 | 2.595 | 13.4 (7.8-23.0) |  | 39.8 | 1.611 | 5.01 (3.90-6.43) |  | 23.7 | 1.473 | 4.36 (3.59-5.31) |  | 31.3 | 1.921 | 6.83 (4.94-9.43) |
| **Secondhand smoke exposure** |  |  |  |  |  |  |  |  |  |  |  |  |  |  |  |
| No | 1.6 |  | 1.00 |  | 3.7 |  | 1.00 |  | 3.8 |  | 1.00 |  | 1.6 |  | 1.00 |
| Yes | 7.3 | 1.132 | 3.10 (1.89-5.10) |  | 10.5 | 0.362 | 1.44 (1.13-1.82) |  | 10.0 | 0.381 | 1.46 (1.27-1.69) |  | 9.3 | 0.971 | 2.64 (1.81-3.86) |
| **Tobacco advertisements exposure** |  |  |  |  |  |  |  |  |  |  |  |  |  |  |  |
| No | 2.0 |  | 1.00 |  | 4.4 |  | 1.00 |  | 5.6 |  | 1.00 |  | 3.5 |  | 1.00 |
| Yes | 6.3 | 0.763 | 2.14 (1.22-3.77) |  | 9.3 | 0.331 | 1.39 (1.17-1.66) |  | 7.9 | -0.086 | 0.92 (0.79-1.06) |  | 4.8 | 0.293 | 1.34 (1.05-1.72) |
| **Being taught about dangers of smoking** |  |  |  |  |  |  |  |  |  |  |  |  |  |  |  |
| Yes | 3.6 |  | 1.00 |  | 7.1 |  | 1.00 |  | 7.4 |  | 1.00 |  | 5.5 |  | 1.00 |
| No | 7.3 | 0.804 | 2.24 (1.37-3.66) |  | 8.6 | 0.211 | 1.24 (1.04-1.46) |  | 7.0 | 0.171 | 1.19 (1.03-1.37) |  | 8.7 | 0.185 | 1.20 (0.98-1.47) |
| **Survey year** |  |  |  |  |  |  |  |  |  |  |  |  |  |  |  |
| 2016-2019 | 2.8 |  | 1.00 |  | 6.6 |  | 1.00 |  | 6.6 |  | 1.00 |  | 3.5 |  | 1.00 |
| 2010-2015 | 5.9 | 0.840 | 2.32 (1.40-3.82) |  | 8.9 | 0.662 | 1.94 (1.55-2.42) |  | 8.6 | -1.247 | 0.29 (0.21-0.40) |  | 8.9 | 1.142 | 3.13 (1.90-5.17) |

All variables listed in the table were introduced into logistic regression models.

OR: odds ratio; CI: confidence interval.

**Table S13. Factors associated with waterpipe use (on ≥1 day during the past 30 days) among adolescents aged 12-16 years by status of cigarette smoking**

| Variable | **Current cigarette smoking** | | |  | **No cigarette smoking** | | |
| --- | --- | --- | --- | --- | --- | --- | --- |
|  | Prevalence (%) | *β* | OR (95% CI) |  | Prevalence (%) | *β* | OR (95% CI) |
| **Sex** |  |  |  |  |  |  |  |
| Girls | 25.3 |  | 1.00 |  | 3.6 |  | 1.00 |
| Boys | 32.8 | 0.128 | 1.14 (0.93-1.39) |  | 4.9 | 0.268 | 1.31 (1.15-1.49) |
| **Age group** |  |  |  |  |  |  |  |
| 12-14 years | 26.7 |  | 1.00 |  | 3.8 |  | 1.00 |
| 15-16 years | 33.7 | 0.146 | 1.16 (0.96-1.40) |  | 4.9 | 0.195 | 1.22 (1.05-1.41) |
| **Parental smoking** |  |  |  |  |  |  |  |
| Neither | 36.0 |  | 1.00 |  | 3.8 |  | 1.00 |
| Father only | 41.5 | 0.112 | 1.12 (0.88-1.42) |  | 7.1 | 0.498 | 1.65 (1.44-1.88) |
| Mother only | 49.5 | 0.177 | 1.19 (0.87-1.63) |  | 17.3 | 1.378 | 3.97 (2.90-5.43) |
| Both | 46.2 | 0.187 | 1.21 (1.33-1.62) |  | 16.2 | 1.117 | 3.06 (2.51-3.72) |
| **Smoking status of closest friends** |  |  |  |  |  |  |  |
| None | 23.7 |  | 1.00 |  | 3.3 |  | 1.00 |
| Some | 24.9 | 0.318 | 1.37 (1.06-1.78) |  | 8.6 | 0.824 | 2.28 (1.98-2.63) |
| Most | 32.1 | 0.753 | 2.12 (1.64-2.74) |  | 11.8 | 1.090 | 2.97 (2.42-3.66) |
| All | 55.7 | 1.127 | 3.09 (2.14-4.45) |  | 18.5 | 1.493 | 4.45 (3.20-6.19) |
| **Secondhand smoke exposure** |  |  |  |  |  |  |  |
| No | 20.1 |  | 1.00 |  | 2.3 |  | 1.00 |
| Yes | 31.8 | 0.361 | 1.44 (1.08-1.91) |  | 5.9 | 0.444 | 1.56 (1.35-1.80) |
| **Tobacco advertisements exposure** |  |  |  |  |  |  |  |
| No | 25.4 |  | 1.00 |  | 2.8 |  | 1.00 |
| Yes | 30.9 | 0.370 | 1.45 (1.13-1.85) |  | 4.7 | 0.171 | 1.19 (1.05-1.34) |
| **Being taught about dangers of smoking** |  |  |  |  |  |  |  |
| Yes | 30.4 |  | 1.00 |  | 4.2 |  | 1.00 |
| No | 29.3 | 0.171 | 1.19 (1.00-1.41) |  | 4.8 | 0.279 | 1.32 (1.15-1.52) |
| **Survey year** |  |  |  |  |  |  |  |
| 2016-2019 | 23.0 |  | 1.00 |  | 3.5 |  | 1.00 |
| 2010-2015 | 39.3 | 0.681 | 1.98 (1.59-2.45) |  | 5.0 | 0.300 | 1.35 (1.12-1.62) |

All variables listed in the table were introduced into logistic regression models.

OR: odds ratio; CI: confidence interval.
